# Biomimetic Nanoplatform for Targeted Rheumatoid Arthritis Therapy: Modulating Macrophage Niches Through Self‐Sustaining Positive Feedback‐Driven Drug Release Mechanisms

**DOI:** 10.1002/advs.202416265

**Published:** 2025-02-22

**Authors:** Huichao Xie, Xiaoyu Huang, Bao Li, Yongfeng Chen, Haoran Niu, Tong Yu, Shimei Yang, Shuxin Gao, Yutong Zeng, Tianzhi Yang, Yan Kang, Keda Zhang, Pingtian Ding

**Affiliations:** ^1^ College of Pharmacy Shenzhen Technology University Shenzhen 518118 China; ^2^ Guangdong Key Laboratory for Biomedical Measurements and Ultrasound Imaging National‐Regional Key Technology Engineering Laboratory for Medical Ultrasound School of Biomedical Engineering Shenzhen University Medical School Shenzhen 518060 China; ^3^ School of Pharmacy Shenyang Pharmaceutical University Shenyang 110016 China; ^4^ College of Pharmacy and Health Sciences Western New England University Springfield MA 01119 USA

**Keywords:** biomimetic nanodelivery platform, gene silencing, guanidinium‐based polymers, prussian blue nanoenzymes, rheumatoid arthritis

## Abstract

The core strategies in treating rheumatoid arthritis (RA) now focus on ameliorating the inflammatory microenvironment and reversing macrophage phenotypes within the joint cavity. This study introduces a co‐delivery system of integrating nanoenzymes and gene therapeutics sequentially modified with guanidinium‐based polymers and macrophage membranes to achieve synergistic therapeutic effects. This co‐delivery system is named MACP siTNF‐α nanoparticles (NPs). MACP siTNF‐α nanoparticles are designed for targeted delivery to the inflamed joint site, where they are preferentially internalized by M1‐type macrophages and efficiently evade lysosomal degradation. Subsequently, the co‐delivery system operates efficiently via a self‐sustaining positive feedback drug release mechanism. The biomimetic nanoplatform reduces reactive oxygen species (ROS) levels and prevents glutathione (GSH) depletion. GSH degrades the polymers to release small interfering RNA (siRNA) and expose the Prussian blue (PB) nanoenzymes, which effectively scavenge ROS and restore GSH levels. This feedback loop significantly enhances the gene silencing capability and ROS scavenging efficiency of the co‐delivery system. In summary, MACP siTNF‐α NPs can reverse macrophage ecological niche in inflammatory soils through the dual mechanism of efficiently inhibiting the expression of tumor necrosis factor‐alpha (TNF‐α) the upstream pathway of the inflammatory response, and eliminating ROS, thus realizing efficient treatment of RA.

## Graphical abstract

Rheumatoid arthritis is an incurable inflammatory autoimmune disease. The cleverly biomimetic nanoplatform integrates nanoenzymes and gene therapeutics and is sequentially modified by guanidinium‐based polymers and macrophage membranes, enhancing the gene silencing capability and reactive oxygen species scavenging efficiency through self‐sustaining positive feedback drug release mechanisms, reversing macrophage ecological niche in inflammatory soils, thus realizing efficient treatment of rheumatoid arthritis.

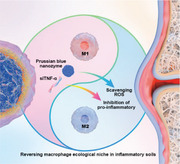

## 1 Introduction

Rheumatoid arthritis is an incurable inflammatory autoimmune disease that predominantly affects small joints.^[^ ^1^ ^]^ Globally, the prevalence of RA is ≈0.5%, with a higher incidence in females, occurring two to three times more frequently than in males.^[^ ^2^ ^]^ The progression of RA can be partitioned into three stages: an initial nonspecific inflammatory stage, a chronic inflammatory phase driven by T‐cell activation in the synovium, and a final tissue damage phase mediated by cytokines such as IL‐1, IL‐6, and TNF‐α. Pro‐inflammatory and bone‐degrading factors associated with immune responses lead to synovial membrane thickening, angiogenesis, muscle atrophy, and the erosion of cartilage and subchondral bone, resulting in joint deformities, dysfunction, and even disability.^[^ ^3^ ^]^ Macrophages exhibit phenotypic plasticity, and the phenotype of infiltrated macrophages in the articular cavity determines the trend of RA.^[^ ^4^ ^]^ Inflammatory soils polarize macrophages toward a pro‐inflammatory M1 phenotype, which promotes monocyte recruitment, synovial fibroblast proliferation, and osteoclast formation by secreting pro‐inflammatory cytokines such as TNF‐α and IL‐6. A promising therapeutic approach for RA involves reducing ROS levels to mitigate oxidative stress, suppressing pro‐inflammatory factors, ameliorating the inflammatory soil, reversing macrophage polarization to the anti‐inflammatory M2 phenotype, and enhancing tissue repair.^[^ ^5^ ^]^

TNF‐α is a pleiotropic cytokine that triggers complex immunoregulatory pathways and serves as a central mediator of various immune‐mediated diseases.^[^ ^6^ ^]^ Chronic overproduction of TNF‐α is a major driver of autoimmune diseases. Both membrane‐bound (mTNF‐α) and soluble (sTNF‐α) TNF forms bind to TNFR1, recruiting proteins such as tumor necrosis factor receptor‐associated death domain (TRADD) and receptor‐interacting serine/threonine‐protein kinase 1 (RIPK1).^[^ ^7^ ^]^ Depending on the ubiquitination status of RIPK1, various downstream pathways can be further activated, primarily the MAPK pathway (including extracellular signal‐regulated kinase (ERK), c‐Jun N‐terminal kinase (JNK), and p38 cascades) and the NF‐κB pathway, ultimately inducing apoptosis, necroptosis, and promoting ROS production.^[^ ^8^ ^]^

**Figure 1:**
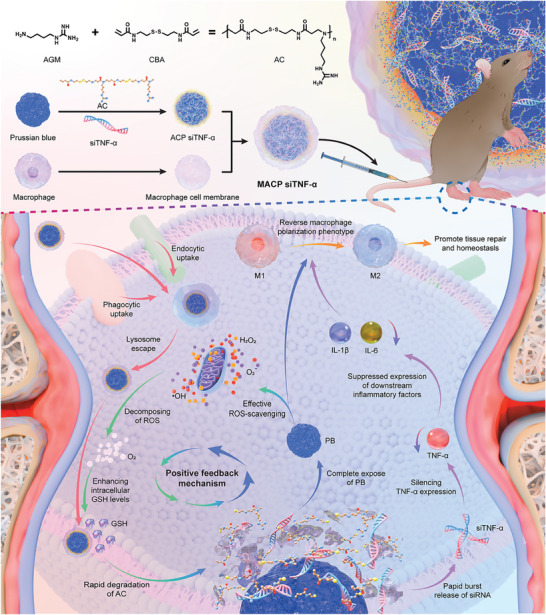
Mechanism of the biomimetic nanodelivery platform (MACP siTNF‐α NPs) for rheumatoid arthritis treatment. The biomimetic nanodelivery platform reverses the macrophage ecological niche in inflammatory environments through a self‐sustaining positive feedback drug release mechanism. This nanoplatform reduces ROS levels and prevents GSH depletion, allowing GSH to degrade the polymers, release siRNA, and expose PB nanoenzymes. The PB nanoenzymes effectively scavenge ROS and restore GSH levels. This mechanism enhances both gene silencing and ROS scavenging efficiency in the co‐delivery system. Briefly, MACP siTNF‐α NPs improve the inflammatory microenvironment in the joint cavity through dual mechanisms: efficiently silencing TNF‐α expression in the upstream inflammatory pathway and eliminating ROS, thereby reversing macrophage polarization and achieving high‐efficiency RA therapy.

Small interfering RNAs, which can theoretically silence the expression of any given gene by degrading mRNAs, have emerged as a powerful signaling pathway‐blocking technology.^[^ ^9^ ^]^ Gene technology offers a means to silence TNF expression, thereby providing sustained inhibition of inflammatory signaling pathways. An ideal platform for in vivo siRNA delivery should ensure blood circulation stability, evade immune clearance, accumulate specifically and sustainably, facilitate endosomal escape, and enable rapid intracellular release. To achieve this, we synthesized low molecular weight guanidinium‐based polymers (referred to as AC, with a molecular weight of 1000–3000 Da) containing disulfide bonds that are rapidly degraded by GSH. AC can adsorb onto the surface of siRNA through electrostatic interactions between guanidino groups and siRNA, shielding the gene from direct exposure to physiological environments, preventing degradation, and responding to GSH degradation to enable the rapid release of the gene (**Figure** **1**).

Under pathological conditions, ROS overexpression can impair antioxidant enzyme activity and deplete intracellular glutathione (GSH), rendering the antioxidant system insufficient to balance excessive ROS levels.^[^ ^10^ ^]^ Nanoenzymes, which are nanomaterials combining the catalytic properties of traditional chemical and biological catalysts, have been widely employed to protect intracellular biomolecules.^[^ ^11^ ^]^ Compared to natural enzymes, nanozymes offer advantages such as lower production costs, prolonged activity, and high catalytic stability. Nanoenzymes are primarily divided into two categories: 1) oxidoreductase family, which includes oxidase (Au),^[^ ^12^ ^]^ peroxidase (CuS),^[^ ^13^ ^]^ catalase (MnO_2_),^[^ ^14^ ^]^ superoxide dismutase (CeO_2_),^[^ ^15^ ^]^ and nitrate reductase (CdS‐Pt)^[^ ^16^ ^]^ and 2) hydrolytic enzyme family, such as phosphatase (MOF‐CeO_2_),^[^ ^17^ ^]^ proteases (CeONP),^[^ ^18^ ^]^ among others. Prussian Blue (PB) is an iron‐based nanoenzyme with the structural formula A[FeIIIFeII(CN)_6_] (A = Na^+^ or K^+^), exhibiting self‐enhancing peroxidase, catalase, and superoxide dismutase activities. Recent research by Zhang et al. has detailed the catalytic mechanism of PB, which catalyzes electron flow from reduced substrates to hydrogen peroxide through a dual‐path mechanism involving both valence and conduction band‐mediated pathways.^[^ ^19^ ^]^ This catalytic activity can be sustained and even slightly enhanced over extended periods.

In this study, we designed and constructed a biomimetic nanoplatform (MACP siTNF‐α NPs) with the primary therapeutic objectives of reducing ROS levels in the joint cavity, inhibiting inflammatory factor expression, improving the inflammatory microenvironment, and reversing macrophage polarization to mitigate RA progression and promote joint tissue repair. The platform consists of nanoenzymes and gene therapeutics sequentially modified with guanidinium‐based polymers and macrophage membranes. The macrophage membrane coating enabled prolonged circulation in vivo, targeted inflammation site delivery, selective uptake by M1‐type macrophages in the joint cavity, and lysosomal escape via the “proton sponge effect” of the guanidinium group. MACP siTNF‐α NPs adsorbed ROS on their surface and catalyzed its decomposition, reducing intracellular ROS levels and alleviating GSH depletion. The disulfide bond‐containing guanidinium‐based polymers were rapidly degraded by GSH, efficiently releasing siRNAs to silence TNF‐α expression. Simultaneously, PB nanoenzymes, as efficient ROS scavengers, were fully exposed within cells, facilitating sustained GSH restoration and further polymer degradation (Figure 1).

This system created a positive feedback mechanism: the biomimetic nanoplatform reduced ROS levels and prevented GSH depletion; GSH degraded the polymers, releasing siRNA and exposing PB nanoenzymes; PB nanoenzymes then scavenged ROS and restored GSH levels. This feedback loop significantly enhanced the gene silencing and ROS scavenging capabilities of the co‐delivery system. In summary, MACP siTNF‐α NPs improved the inflammatory microenvironment within the joint cavity through dual mechanisms – efficient TNF‐α silencing in the inflammatory pathway and ROS elimination. These actions successfully reversed macrophage polarization and achieved high therapeutic efficiency for RA.

## 2 Results and Discussion

### 2.1 Preparation and Characterization of MACP siTNF‐α NPs

**Figure 2:**
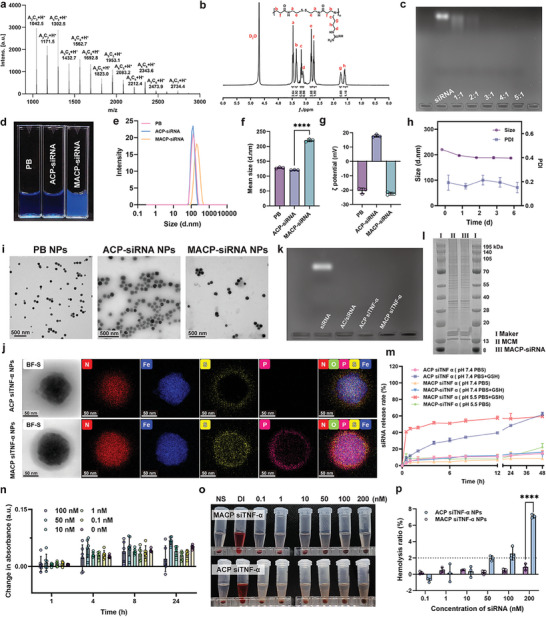
Preparation and physicochemical characterization of MACP siTNF‐α NPs. a) MALDI‐TOF‐MS spectra of AC. b) ^1^H‐NMR spectra of AC. c) Agarose gel electrophoresis of AC/siRNA complexes. d) Representative images of aqueous dispersions of PB NPs, ACP siTNF‐α NPs, and MACP siTNF‐α NPs. e) DLS analysis of hydrodynamic diameters, *n* = 3. f) Particle size and g) zeta potential of PB NPs, ACP siTNF‐α NPs, and MACP siTNF‐α NPs, *n* = 3. h) Colloidal stability of MACP siTNF‐α NPs over time, *n* = 3. i) Representative TEM images and j) elemental mapping of ACP siTNF‐α NPs and MACP siTNF‐α NPs. k) Agarose gel electrophoresis showing siRNA association ability of AC, ACP siRNA NPs, and MACP siRNA NPs. l) SDS‐PAGE and Coomassie Brilliant Blue staining for protein band analysis of MACP siTNF‐α NPs. m) In vitro cumulative release analysis of ACP siTNF‐α NPs and MACP siTNF‐α NPs, *n* = 3. n) Serum stability analysis of MACP siTNF‐α NPs at different concentrations, *n* = 6. o) Hemolysis images and p) Hemolysis rate analysis of MACP siTNF‐α NPs and ACP siTNF‐α NPs, *n* = 3. Data are presented as mean ± SD, with comparisons in (f, p) by one‐way ANOVA. *p < 0.05, **p < 0.01, ***p < 0.001, ****p < 0.0001.

Based on previous experience, low molecular weight polyamidoamine polymers containing disulfide bonds (referred to as AC) were synthesized by Michael addition polymerization.^[^ ^20^ ^]^ The resulting products were verified using ^1^H‐NMR and MALDI‐TOF‐MS. The molecular weight distribution of AC was determined by Matrix‐assisted Laser Desorption/Ionization‐Time‐of‐Flight MS (MALDI‐TOF‐MS), with the molecular weight calculated as exact mass = 130.12*x* + 260.07*y*, expressed as A*_x_* C*_y_*. Results indicated that the molecular weight of A*_x_* C*_y_* predominantly fell within the range of 1000–3000 Da, with A_2_C_3_, A_4_C_3_, and A_4_C_4_ being the main forms observed (**Figure** **2**a). This low molecular weight facilitates rapid degradation in a reducing environment, enabling efficient gene release. The ^1^H‐NMR (400 MHz, D_2_O) spectrum for AC (Figure 2b) showed the following chemical shifts: *δ* = 3.47 (−SSCH2 ***CH2*** NH−), *δ* = 3.35 (−COCH2 ***CH2*** N−), *δ* = 3.18 (−CH2N ***CH2*** CH2CH2CH2−), *δ* = 3.12 (−***CH2*** N3H4), *δ* = 2.81 (−SS ***CH2*** CH2−), *δ* = 2.72 (−NHCO ***CH2*** CH2N−), *δ* = 1.75 (−NCH2 ***CH2*** CH2−), δ = 1.60 (−NCH2CH2 ***CH2***−). Although the molecular weight of AC is significantly smaller than that of siRNA, its guanidinium group binds to the surface of siRNA through electrostatic interactions, forming a stable complex. Agarose gel electrophoresis (Figure 2c) confirmed that siRNA could be stably loaded at a mass ratio of 3:1 (AC to siRNA).

Hydrophilic Prussian Blue (PB) nanoenzymes, modified with poly(vinylpyrrolidone) (PVP, K30), were synthesized using a hydrothermal method. These were then co‐incubated with AC and siRNA in a one‐pot process to prepare ACP siTNF‐α NPs. Finally, MACP siTNF‐α NPs with targeted inflammation properties were created by applying a macrophage membrane coating onto the nanoparticles via sonication. Photographs of aqueous dispersions of PB NPs, ACP siTNF‐α NPs, and MACP siTNF‐α NPs are shown in Figure 2d. The nanoparticle characteristics were analyzed by dispersing PB NPs, ACP siTNF‐α NPs, and MACP siTNF‐α NPs in deionized water and assessing them using dynamic light scattering (DLS). DLS analysis (Figure 2e,f) revealed that hydrophilic PB NPs had a particle size of 127.80 ± 2.10 nm with a narrow size distribution (PDI = 0.064). The particle size of ACP siTNF‐α NPs was slightly smaller at 120.30 ± 0.78 nm, likely due to the PVP on PB's surface being in an extended state in aqueous solution; the electrostatic interactions between AC and PVP caused polymer chain shrinkage, compressing the thickness of the modified layer. ACP siTNF‐α NPs also had a narrow size distribution (PDI = 0.023), and the surface potential shifted from ‐20.50 ± 1.96 mV to +17.76 ± 0.92 mV (Figure 2g), indicating uniform coating of AC and AC‐siRNA complexes on the PB surface. For MACP siTNF‐α NPs, the particle size increased to 220.33 ± 4.30 nm with a PDI of less than 0.3, suggesting good size homogeneity. After biomimetic coating, the surface potential shifted to –22.52 ± 1.06 mV, which aligned closely with the membrane potential, confirming successful macrophage membrane modification. Additionally, MACP siTNF‐α NPs demonstrated stable colloidal properties over six days (Figure 2h).

The microscopic morphology of PB NPs, ACP siTNF‐α NPs, and MACP siTNF‐α NPs was observed using transmission electron microscopy (TEM). TEM images (Figure 2i) revealed that PB NPs, ACP siTNF‐α NPs, and MACP siTNF‐α NPs appeared as monodispersed spheres with uniform sizes. The particle sizes measured by dynamic light scattering (DLS) reflected the hydrodynamic size in aqueous dispersion, while TEM‐measured sizes were slightly smaller due to nanoparticle shrinkage in the dry state.^[^ ^21^ ^]^ Additionally, elemental mapping analysis was conducted using energy‐dispersive X‐ray spectroscopy (EDS) in scanning transmission electron microscopy (STEM) mode. Bright‐field STEM (BF‐S) imaging results (Figure 2j) showed that MACP siTNF‐α NPs displayed a well‐defined core‐shell structure. A distinct sulfur (S) element coating was observed on the surface of ACP siTNF‐α NPs, indicating the uniform coating of AC and AC‐siRNA complexes on the PB core. Phosphorus (P) element coatings were seen on MACP siTNF‐α NPs, with a coating thickness of ≈15–20 nm, consistent with the thickness of a cell membrane. This confirmed that macrophage membranes had successfully provided complete biomimetic modification on the nanoparticle surfaces.

The encapsulation efficiency (EE%) and drug loading rate (DL%) of MACP siTNF‐α NPs were determined using fluorescence spectrophotometry with FAM‐labeled siRNA. The encapsulation efficiency and drug loading for siRNA in MACP siTNF‐α NPs were 95.92% ± 0.65% and 3.262‰ ± 0.64‰, respectively. Agarose gel electrophoresis was performed to assess the siRNA loading capacity of AC siTNF‐α, ACP siTNF‐α NPs, and MACP siTNF‐α NPs. As shown in Figure 2k, all formulation forms achieved stable siRNA loading without leakage at a mass ratio of 100:20:1 (AC:PB:siRNA).

Evidence suggests that macrophage membrane‐encapsulated nanocarriers can evade immune surveillance and possess chemotactic capabilities directed at sites of chronic inflammation. We further evaluated the coating quality of macrophage membranes on MACP siTNF‐α NPs using SDS‐PAGE. As shown in Figure 2l, membrane proteins were successfully transferred to the nanoparticle surface along with the lipid membranes during preparation, a critical feature for enabling inflammation‐targeted functionality in vivo.

### 2.2 In Vitro siRNA Release Behavior of MACP siTNF‐α NPs

The release behavior of ACP siTNF‐α NPs and MACP siTNF‐α NPs was evaluated in different release media (PBS at pH 7.4, PBS at pH 5.5, PBS at pH 7.4 + GSH, and PBS at pH 5.0 + GSH) to assess the in vitro release of siRNA (Figure 2m). In PBS at pH 7.4, the cumulative release rates of ACP siTNF‐α NPs and MACP siTNF‐α NPs were low, with no initial burst observed; after 48 h, cumulative release rates were 14.76% and 8.56%, respectively, suggesting stability in storage and circulation. The intracellular GSH concentration (1–10 mM) was substantially higher than extracellular concentrations, providing a mechanism for selective intracellular release of drugs and genes.^[^ ^22^ ^]^ In PBS at pH 7.4 + GSH, the release rate of ACP siTNF‐α NPs increased significantly. The cumulative release reached 30.11% within 6 h and 61.67% at 48 h, confirming the reduction sensitivity of AC. The disulfide bonds in AC were rapidly degraded by GSH, enabling efficient siRNA release. In contrast, the release rate of MACP siTNF‐α NPs in PBS at pH 7.4 + GSH did not increase significantly, likely due to the complete biomimetic coating, which protected siRNAs from degradation in complex enzyme environments and prevented leakage and rapid release. Further investigation of MACP siTNF‐α NPs in PBS at pH 5.5 + GSH revealed that the cumulative release rate rose sharply to 44.52% within 1 hour. This finding suggests that MACP siTNF‐α NPs can disrupt the biomimetic membrane through the proton sponge effect, facilitating lysosomal escape and enabling efficient siRNA release.

### 2.3 Blood Compatibility of MACP siTNF‐α NPs

Serum stability was assessed, as shown in Figure 2n. No significant change in absorbance was observed for MACP siTNF‐α NPs across a range of siRNA concentrations from 0 to 100 nM over 24 h, indicating minimal interaction between the components of the co‐incubation system. Hemolysis rates were evaluated as depicted in Figure 2o,p. After centrifugation, no significant hemoglobin leakage was detected in the supernatants of MACP siTNF‐α NPs at siRNA concentrations between 0.1 and 200 nM, with all hemolysis rates remaining below 2%. This demonstrates that MACP siTNF‐α NPs did not significantly impact the physiological integrity of red blood cells within siRNA concentrations of 0.1–100 nM, indicating excellent hemocompatibility for intravenous injection.

### 2.4 Biocompatibility of MACP siTNF‐α NPs

**Figure 3:**
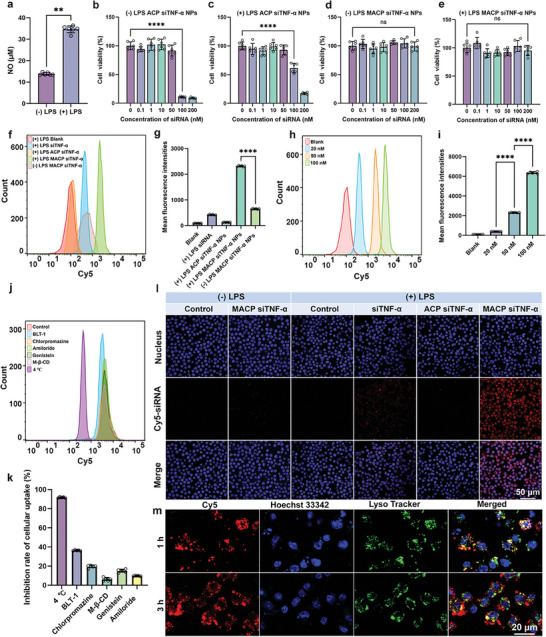
Cellular uptake and lysosomal escape. a) Nitrous oxide (NO) levels in RAW 264.7 cells with or without LPS stimulation, *n* = 6. b–e) Cytotoxicity of ACP siTNF‐α NPs and MACP siTNF‐α NPs against RAW 264.7 cells with or without LPS stimulation over 24 h, *n* = 6. f,g) Flow cytometry analysis of cellular uptake in RAW 264.7 cells with or without LPS stimulation, respectively, *n* = 6. h,i) Flow cytometry analysis of MACP siTNF‐α NP uptake by M1 macrophages, *n* = 6. j,k) Flow cytometry analysis of cellular uptake mechanisms with different inhibitors, *n* = 5. l) Confocal microscopy images showing cellular uptake in RAW 264.7 cells with or without LPS stimulation, *n* = 3. m) Confocal microscopy images of lysosomal escape, *n* = 3. Data are shown as means ± SD, data in (a) were compared by Student's t‐test, and data in (b, c, d, e, g, i) were compared by one‐way ANOVA. *p < 0.05, **p <0.01, ***p <0.001, and ****p < 0.0001.

As innate immune cells, macrophages can polarize into distinct phenotypes in response to various physiological and pathological pathological conditions both in vivo and in vitro.^[^ ^23^ ^]^ Lipopolysaccharide (LPS) is recognized by the surface receptor TLR4 on macrophages, which activates the JAK2/STAT1 pathway, thereby inducing macrophage polarization.^[^ ^24^, ^25^ ^]^ To establish a pro‐inflammatory macrophage model, RAW 264.7 cells were polarized into M1‐type macrophages using LPS. Nitric oxide (NO) production in the culture supernatants was measured using Griess reagent to confirm the establishment of the cell model. Macrophages exposed to LPS showed a significant increase in NO generation, verifying the successful establishment of the M1 macrophage inflammation model (**Figure** **3**a).

Minimizing the cytotoxicity of the delivery system is essential to ensure macrophage survival and maintain the functionality of the formulation. The cytotoxicity of ACP siTNF‐α NPs and MACP siTNF‐α NPs on macrophages in different states (M0 and M1) was assessed using the CCK8 assay. As shown in Figure 3b–e, at siRNA concentrations below 50 nM, both ACP siTNF‐α NPs and MACP siTNF‐α NPs exhibited negligible cytotoxicity. However, at an siRNA concentration of 100 nM, ACP siTNF‐α NPs caused significant cytotoxicity in both macrophage states (M0: 10.70%, M1: 61.25%), whereas MACP siTNF‐α NPs showed no significant cytotoxicity across the examined concentration range. These results indicate that the biomimetic membrane modification strategy effectively improves the safety of the drug delivery system. The natural compatibility between macrophage membranes and macrophages enhances cellular safety for MACP siTNF‐α NPs.

### 2.5 Intracellular Delivery and Rapid Lysosome Escape of MACP siTNF‐α NPs

Efficient cellular uptake is critical for effective siRNA delivery. The uptake of MACP siTNF‐α NPs by macrophages was first quantitatively assessed using flow cytometry. As shown in Figure 3f,g, M0‐type macrophages in an inactive state displayed low uptake capacity, with no significant uptake of MACP siTNF‐α NPs observed. However, M1‐type macrophages exhibited significantly enhanced uptake of MACP siTNF‐α NPs (****p < 0.0001), with uptake efficiency increasing by 3.56‐fold compared to M0‐type macrophages. This result suggested that macrophage membrane‐modified MACP siTNF‐α NPs were selectively taken up by M1‐type macrophages.

Furthermore, as shown in Figure 3h,i, the uptake efficiency in M1‐type macrophages displayed a clear concentration dependence. When the siRNA concentration was raised from 20 to 100 nM, uptake efficiency increased 16.31‐fold (mean fluorescence intensity: 389.50 ± 4.18 versus 6352.17 ± 100.49). Flow cytometry results indicated that M1‐type macrophages could take up small amounts of siRNA, potentially due to increased phagocytosis mediated by scavenger receptors in activated M1‐type macrophages. Interestingly, no effective uptake of ACP siTNF‐α NPs by M1‐type macrophages was observed, likely due to several factors. ACP siTNF‐α NPs reduced ROS and elevated GSH levels within the co‐incubation system, causing siRNA release through AC degradation before cellular uptake. Additionally, guanidino fragments from degraded AC bound electrostatically to LPS on the siRNA surface, further reducing cellular uptake efficiency. These results highlighted the importance of biomimetic membrane modification in stabilizing siRNA and enhancing the uptake efficiency of MACP siTNF‐α NPs.

Confocal laser scanning microscopy (CLSM) further visualized the uptake of MACP siTNF‐α NPs by M0 and M1 macrophages, with findings consistent with flow cytometry data (Figure 3l). M0‐type macrophages showed no significant uptake of MACP siTNF‐α NPs, while M1‐type macrophages demonstrated efficient, selective uptake. These results suggested that MACP siTNF‐α NPs could effectively avoid nonspecific macrophage uptake in circulation and prevent rapid clearance in vivo. Activated M1‐type macrophages, abundant in rheumatoid arthritis‐affected areas, especially at the cartilage‐pannus junction, were targeted by MACP siTNF‐α NPs at inflammation sites, promoting the enrichment of therapeutic agents in target areas and enhancing drug delivery efficacy.

To investigate the uptake mechanism of MACP siTNF‐α NPs by M1‐type macrophages, a series of endocytosis and phagocytosis inhibitors (targeting scavenger receptors) were preincubated in the co‐incubation system. As shown in Figure 3j,k, the inhibition of MACP siTNF‐α NP uptake by M1‐type macrophages reached 91.60% following treatment at 4 °C, indicating that the nanoparticle uptake process was energy‐dependent. Among the inhibitors tested, block lipid transport‐1 (BLT‐1) produced the most substantial inhibition, with an inhibition rate of 36.34%, suggesting that scavenger receptor‐mediated phagocytosis was the primary pathway for M1 uptake of MACP siTNF‐α NPs. The chlorpromazine group (19.43%), genistein group (14.76%), amiloride group (9.75%), and methyl‐β‐cyclodextrin group (6.16%) exhibited varying degrees of endocytosis inhibition. These results suggested that the uptake and internalization of MACP siTNF‐α NPs were facilitated by both scavenger receptor‐mediated phagocytosis and multiple endocytic pathways, including clathrin‐mediated endocytosis, macropinocytosis, caveolin‐mediated endocytosis, and lipid raft‐mediated endocytosis after RAW cells were induced by LPS to differentiate from M0 to M1.

Efficient gene silencing requires that siRNA rapidly and effectively escapes from endosomes or lysosomes to form RNA‐induced silencing complexes (RISCs) in the cytoplasm alongside Argonaute (AGO) proteins.^[^ ^26^, ^27^ ^]^ The lysosomal escape ability of MACP siTNF‐α NPs following cellular uptake was further examined using CLSM with Cy5‐labeled siTNF‐α. As shown in Figure 3m, after 1 h of co‐incubation with M1‐type macrophages, partial separation of the Cy5 fluorescence signal from Lyso Tracker Green was observed. After 3 h, the overlap between Cy5 and Lyso Tracker Green signals was further reduced, indicating a widespread separation in the observation field. These results demonstrated that MACP siTNF‐α NPs efficiently escaped from lysosomes and released siRNA shortly after being internalized by M1‐type macrophages. The likely mechanism for this lysosomal escape is the high buffering capacity of the guanidinium functional group in the AC structure, which induces an influx of chloride ions and water into the lysosome via the proton sponge effect. This increases osmotic pressure, leading to lysosomal membrane disruption and enabling siRNA escape.

### 2.6 In Vitro Anti‐Inflammatory Activities of MACP siTNF‐α NPs

Nanoenzymes exhibit enzymatic activity by effectively mimicking the catalytic sites of natural enzymes or incorporating multivalent elements for reactions. They offer higher catalytic stability, easier modification, and lower manufacturing costs than traditional catalases, such as proteases.^[^ ^28^ ^]^ PB nanoenzymes efficiently adsorbed reactive oxygen radicals on their surface, aided by their large specific surface area. This facilitated substrate decomposition via electron transfer, following a mechanism similar to the Fenton reaction, where the Fe valence state shifts through electron transfers between H_2_O_2_, superoxide anion (O_2_·), and hydroxyl radicals (·OH).^[^ ^29^ ^]^

**Figure 4:**
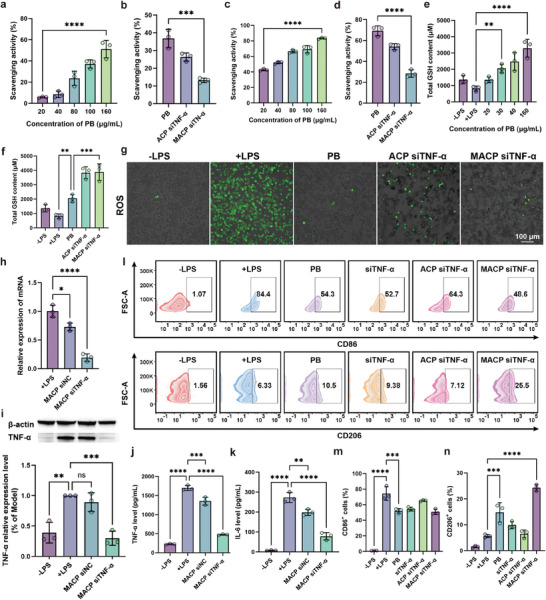
Nanoenzymatic activity, gene silencing efficiency, and in vitro immunoassays. a,c) Quantification of catalase and superoxide dismutase activities of PB NPs at various concentrations, *n* = 3. b,d) Quantification of catalase and superoxide dismutase activities of PB NPs, ACP siTNF‐α NPs, and MACP siTNF‐α NPs, with an equivalent PB concentration of 100 µg mL^−1^, *n* = 3. e) Quantification of GSH levels in macrophages treated with different concentrations of PB, *n* = 3. f) Quantification of GSH levels in macrophages treated with PB NPs, ACP siTNF‐α NPs, and MACP siTNF‐α NPs, with an equivalent PB concentration of 100 µg mL^−1^, *n* = 3. g) Fluorescence microscopy images showing the reduction of ROS levels in macrophages treated with PB NPs, ACP siTNF‐α NPs, and MACP siTNF‐α NPs (PB concentration 100 µg mL^−1^), *n* = 3. h) RT‐qPCR analysis of mTNF‐α expression, *n* = 3. i) Protein expression levels of TNF‐α detected by Western blot, with quantification of TNF‐α expression relative to β‐actin. TNF‐α expression in activated RAW 264.7 cells was set to 100%, *n* = 3. Levels of j) TNF‐α and k) IL‐6 in cell supernatants, determined by ELISA, *n* = 3. l) Flow cytometry analysis of macrophage phenotypes, *n* = 3. m, n) Quantification of CD86^+^ (M1 type) and CD206^+^ (M2 type) macrophages, *n* = 3. Data are presented as mean ± SD, with comparisons in (a, b, c, d, e, f, h, i, j, k, m, n) performed by one‐way ANOVA. *p < 0.05, **p < 0.01, ***p < 0.001, ****p < 0.0001.

Hydrogen peroxidase (CAT) and superoxide dismutase (SOD) activities of the formulation were measured using an assay kit according to the manufacturer's instructions. As shown in **Figure** **4**a, PB nanoparticles exhibited efficient hydrogen peroxide scavenging in a concentration‐dependent manner. Increasing the PB concentration by 5‐fold (from 20 to 100 µg mL^−1^) improved clearance by 6.41‐fold (5.79% vs 37.12%, ****p < 0.0001). Figure 4c shows that PB also demonstrated efficient SOD activity, achieving a superoxide anion scavenging rate of 69.21% at 100 µg mL^−1^, providing a foundation for reducing inflammatory levels of RA by eliminating ROS in vivo. The CAT activity of PB was retained effectively after layer‐by‐layer surface modification (Figure 4b), with hydrogen peroxide scavenging rates for ACP siTNF‐α NPs and MACP siTNF‐α NPs recorded at 26.30% and 13.17%, respectively. Similarly, the superoxide anion scavenging rates for ACP siTNF‐α NPs and MACP siTNF‐α NPs were 54.03% and 28.45%, respectively (Figure 4d). These results indicated that PB served as an efficient ROS scavenger and that MACP siTNF‐α NPs could directly exhibit CAT and SOD activities.

GSH, as a natural antioxidant in the body, plays a key role in scavenging excess free radicals to maintain physiological homeostasis.^[^ ^30^ ^]^ GSH is severely depleted within pro‐inflammatory macrophages, so restoring GSH levels and the overall activity of the GSH system is essential to improve macrophage function and regulate polarization patterns.^[^ ^31^ ^]^ In addition, it is critical to elevate the level of GSH in macrophages for efficient degradation of AC, which is a key step to achieve rapid drug release from the nanoplatform. We investigated the impact of PB nanoenzymes and MACP siTNF‐α nanoparticles treatment on intracellular GSH levels in M1‐type macrophages. As demonstrated in Figure 4e, PB nanoenzymes significantly increased intracellular GSH levels in a concentration‐dependent manner. At the equivalent PB concentration, GSH levels were higher in the MACP siTNF‐α NP group than in the PB group (***p < 0.001), which may be due to a combined action of more efficient cellular uptake efficiency and gene silencing effect of siTNF‐α (Figure 4f). In conclusion, MACP siTNF‐α NP and PB efficiently restored GSH levels in M1 macrophages, which ensured the efficient operation of the self‐constructed positive feedback drug release mechanism and facilitated further inflammation elimination and reversal of macrophage polarization.

High levels of mitochondrial ROS are considered a key factor in hindering the repolarization of M1‐type macrophages; therefore, effective ROS clearance within macrophages has become a core indicator for evaluating the anti‐inflammatory properties of the formulation.^[^ ^32^ ^]^ The ROS scavenging ability of MACP siTNF‐α NPs in an LPS‐induced M1‐type macrophage model was assessed using the 2′,7′‐dichlorofluorescein diacetate (DCFH‐DA) probe. As shown in Figure 4g, unactivated M0‐type macrophages produced low ROS levels. When stimulated by LPS, macrophages underwent a metabolic shift from oxidative phosphorylation to glycolysis, triggering oxidative stress through the NADPH oxidase (NOX) 2 enzyme and resulting in a substantial increase in intracellular ROS.

PB was internalized by M1‐type macrophages through active phagocytosis, where it effectively exerted its nanoenzymatic activity and significantly reduced intracellular ROS levels. Although siTNF‐α from ACP siTNF‐α NPs had low uptake efficiency in M1‐type macrophages, ACP siTNF‐α NPs still displayed efficient ROS scavenging. This might have been due to extracellular degradation of AC by GSH, which exposed PB, allowing further uptake and intracellular ROS scavenging. While the CAT and SOD activities of MACP siTNF‐α NPs in vitro were lower than those of PB alone, they demonstrated a potent capacity to scavenge intracellular ROS directly. This effect was largely attributed to the high intracellular uptake efficiency of MACP siTNF‐α NPs and adequate exposure to PB. The results further suggested that the formulation was capable of fully exposing PB in the cytoplasm through the combined action of lysosomal enzymes and GSH‐mediated degradation of AC, which enhanced CAT and SOD activities. This created a positive feedback loop between ROS elimination and GSH elevation.

### 2.7 In Vitro Gene Silencing Efficiency

The gene silencing efficiency of MACP siTNF‐α NPs in M1‐type macrophages was evaluated using quantitative real‐time PCR (RT‐qPCR), Western blotting (WB), and enzyme‐linked immunosorbent assay (ELISA). As shown in Figure 4h, MACP siTNF‐α NPs achieved an 80.79% reduction in TNF mRNA expression, significantly lower than that observed in the LPS‐induced model group (****p < 0.0001). The MACP siNC NPs group exhibited a lower silencing level (27.42%), likely due to PB's ability to partially reduce TNF mRNA expression through ROS elimination. Figure 4i illustrates that TNF protein expression was significantly elevated in the LPS‐induced model group, while the MACP siTNF‐α NPs group demonstrated a notable reduction in protein levels compared to the model group (***p < 0.001). TNF‐α levels in the supernatant of the co‐incubation system were quantified via ELISA, and the results (Figure 4j) aligned with the WB findings. Following LPS stimulation, TNF‐α secretion by M0 macrophages increased 7.54‐fold. Treatment with MACP siTNF‐α NPs reduced TNF‐α secretion by 71.96% in M1‐type macrophages. These findings indicated that MACP siTNF‐α NPs functioned as an efficient siRNA delivery system, achieving substantial silencing of TNF‐α secretion in M1‐type macrophages.

### 2.8 In Vitro Immunologic Assays

To further validate the ability of MACP siTNF‐α NPs to inhibit the secretion of additional inflammatory factors in M1 macrophages, we evaluated changes in the content of the pro‐inflammatory cytokine IL‐6 in the supernatant (Figure 4k). The IL‐6 concentration in the LPS‐induced model group was as high as 272.78 pg mL^−1^, which was reduced to 78.51 pg mL^−1^ in the MACP siTNF‐α NPs group (****p < 0.0001). These changes in pro‐inflammatory cytokine levels indicated that MACP siTNF‐α NPs effectively reduced inflammatory factors through the synergistic actions of ROS elimination and TNF‐α silencing.

RA progression is closely linked to an imbalance between M1 and M2 synovial macrophages, with the repolarization of pro‐inflammatory M1 macrophages to an anti‐inflammatory M2 phenotype being a key strategy for RA mitigation.^[^ ^4^, ^33^ ^]^ To quantitatively assess the effect of MACP siTNF‐α NPs on M1/M2 polarization, we analyzed their surface markers (M1: CD86, M2: CD206) using flow cytometry (Figure 4l–n). Both CD86 and CD206 levels were low on the surface of uninduced M0 macrophages. LPS‐stimulated macrophages showed a CD86^+^ cell rate of 74.4% and an M2/M1 ratio of 0.076, indicating a strong tendency toward pro‐inflammatory M1 polarization. The free siRNA group exhibited a mild tendency to induce M2 polarization in macrophages. Compared to the LPS‐induced group, the PB group showed a 9.12% increase in CD206^+^ cell rate and an M2/M1 ratio of 0.28, suggesting that PB could promote partial reversal of M1‐type to M2‐type macrophages via ROS scavenging. The MACP siTNF‐α NPs group demonstrated the most effective M2 conversion, with the CD86^+^ cell rate reduced to 50.9%, an M1 repolarization rate of 31.59%, a CD206^+^ cell rate increased to 24.27%, and an M2 conversion rate of 79.23%. The MACP siTNF‐α NPs group exhibited an M2/M1 ratio of 0.48, which was twice that of the LPS‐induced group, 1.68‐fold higher than the PB group, and 4.81‐fold higher than the ACP siTNF‐α NPs group. In summary, our study demonstrated that MACP siTNF‐α NPs were selectively taken up by M1‐type macrophages and exerted potent anti‐inflammatory effects through the synergistic actions of nanoenzymes scavenging ROS and siRNA silencing TNF expression, thereby effectively regulating M1/M2 macrophage polarization.

### 2.9 In Vivo Biodistribution Analyses

A collagen‐induced arthritis (CIA) model in mice was established using 8‐week‐old male DBA/1J mice, with an emulsion prepared from bovine type II collagen and complete Freund's adjuvant as an inducer.^[^ ^34^ ^]^ We investigated the therapeutic efficiency, mechanism of action, and safety of MACP siTNF‐α NPs for RA treatment in vivo. First, we assessed the ability of MACP siTNF‐α NPs to accumulate in the inflammatory region (foot) during blood circulation using small animal in vivo imaging, with healthy mice serving as a control group and CIA model mice as the experimental group. CIA and healthy mice received equal doses of DiR‐labeled MACP siTNF‐α NPs (siRNA 1 mg kg^−1^ bw, PB 20 mg kg^−1^ bw) via tail vein injection, and imaging was performed using the IVIS Imaging System. Fluorescent signals were analyzed with Micro‐Manager software.

**Figure 5:**
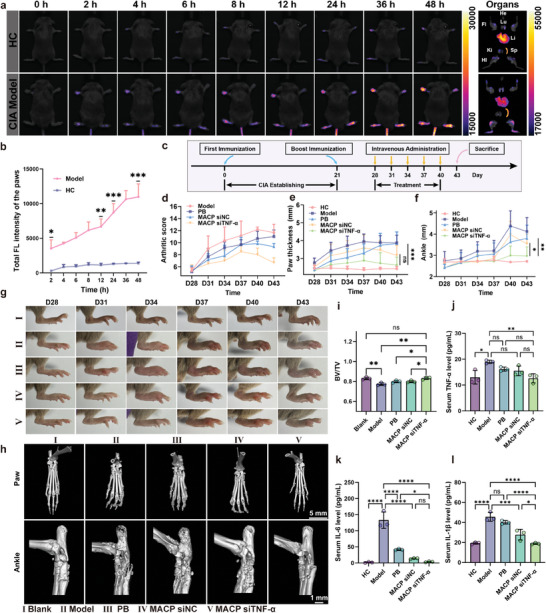
Validation of targeting inflammatory regions and in vivo pharmacodynamic evaluation. a) In vivo fluorescence images of healthy mice and RA model mice following tail vein injection of MACP siTNF‐α NPs, *n* = 3. b) Quantitative analysis of total fluorescence intensity in the paw region of mice, *n* = 3. c) Protocols for CIA model establishment and treatment administration in vivo. Collagen‐induced arthritis in DBA/1 mice serves as an experimental model for human rheumatoid arthritis, with treatment beginning 28 days after the initial immune stimulation. d) Clinical scoring curves, e) hind paw mean thickness, and f) ankle thickness progression over time, *n* = 6. g) Representative photos showing changes in hind paw appearance over time, *n* = 6. h) Representative micro‐CT images and i) normalized bone volume/tissue volume ratios (BV/TV) of the hind paw and ankle joints in CIA mice under different treatments, *n* = 3. Serum levels of j) TNF‐α, k) IL‐6, and l) IL‐1β in CIA mice at treatment endpoints, *n* = 3. Data are presented as mean ± SD, with comparisons in (i–l) conducted by one‐way ANOVA. *p < 0.05, **p < 0.01, ***p < 0.001, ****p < 0.0001.

The results, shown in **Figure** **5**a,b, indicated that a pronounced fluorescence signal appeared in the paws of the CIA model group 2 h postadministration, with fluorescence intensity 35.12 times higher than that of the healthy group. By 48 h postadministration, the fluorescence signal in the paws of the CIA model group continued to increase, reaching a total intensity 3.1 times greater than that observed at 2 h, demonstrating efficient targeting and sustained accumulation of MACP siTNF‐α NPs at the inflammation site. In healthy mice, no significant accumulation was observed in the foot even at 24 h postadministration, with only a minor fluorescence response at 36 h. These findings suggested that MACP siTNF‐α NPs specifically targeted the inflammatory region rather than the organ (paws) in general. The immunoemulsion was injected subcutaneously into the root of the tail of the mice. As a result, the root of the tail in the model group exhibited an inflammatory response. Thus, the accumulation of fluorescent signals was observed at the root of the tail in the model group. Imaging of isolated organs revealed a significant accumulation of MACP siTNF‐α NPs in the knees, ankles, and paws of CIA model mice. The liver and spleen were identified as the primary organs for nanoparticle capture and metabolism. As expected, free MACP siTNF‐α NPs exhibited greater accumulation in the liver and spleen than in other organs. Notably, the formulation's accumulation in the liver and spleen of the CIA model group was significantly lower than in the healthy group, suggesting that in the presence of inflammation, MACP siTNF‐α NPs effectively evaded reticuloendothelial system (RES) phagocytosis and preferentially accumulated in the inflammatory region.

### 2.10 In Vivo Therapeutic Efficacy of MACP siTNF‐α NPs in CIA Mice

The timeline for establishing the CIA animal model and the treatment protocol is illustrated in Figure 5c. The first immunization was performed by injecting 100 µL of an emulsion made from bovine type II collagen and completing Freund's adjuvant. Three weeks later, a booster immunization was administered with 100 µL of an emulsion prepared from bovine type II collagen and incomplete Freund's adjuvant. The development of paw inflammation in the mice was closely monitored thereafter. Mild swelling of the hind paws was observed 28 days after the initial immunization, marking the therapeutic starting point for evaluating the in vivo efficacy of the formulation for RA. The CIA model mice were randomly assigned to four groups: model, PB, MACP siNC, and MACP siTNF‐α groups, while healthy mice served as a blank control group. Tail vein administration was performed five times, with dosing intervals of every 3 days.

The progression of rheumatoid arthritis and the therapeutic effect of the formulation were evaluated macroscopically by clinically scoring paw inflammation, measuring hind paw thickness, and tracking ankle width over time (Figure 5d–f). The clinical scores were graded on a scale of 0–4, all four paws were scored, and the maximal clinical score per mouse was 16.^[^ ^35^, ^36^ ^]^ Joint swelling in RA reflects synovial inflammation due to immune activation, and the clinical inflammation scores over time are shown in Figure 5d, with representative photographs of erythema progression in the right hind limb of mice over time (Figure 5g). The CIA model group displayed a marked increase in clinical scores from day 28 to 37, with visible erythema, severe swelling, and limb stiffness in the ankles, feet, and digits. The PB group showed a slightly slower increase in inflammation compared to the model group, indicating that intravenous PB, a potent nanoenzyme, alleviated inflammation to a certain extent. However, these mice still exhibited erythema and moderate swelling from the ankle to the metatarsal joints.

In the MACP siNC group, inflammation progression was further delayed, likely due to macrophage membrane biomimetic modification, which enhanced treatment efficacy by increasing PB accumulation in the limbs. The MACP siTNF‐α group demonstrated the most significant delay in limb inflammation and showed a gradual decrease in clinical inflammation scores after day 37, with limbs returning to normal without erythema or swelling. At the observation endpoint (day 43), paw thickness in the model group was 1.6 times that of the healthy group, and ankle thickness was 1.52 times that of the healthy group, indicating severe swelling. Both the PB and MACP siNC groups showed some reduction in swelling progression, but therapeutic effects were limited. The foot thickness and ankle width in the MACP siTNF‐α group, however, were restored to levels comparable to the healthy group. The ankle width in the MACP siTNF‐α group was significantly different from that of both the PB group (**p < 0.01) and MACP siNC group (*p < 0.05), as was the foot thickness. Compared to the model group, the MACP siTNF‐α group showed a 32.19% reduction in foot thickness and a 27.88% reduction in ankle width, demonstrating a strong ability to delay foot swelling progression.

Additionally, bones in the foot and ankle were imaged using microcomputed tomography (micro‐CT) to assess the extent of bone destruction. Figure 5h presents 3D reconstructed images of representative micro‐CT scans. The ankle joints of mice in the healthy group appeared structurally intact with smooth bone surfaces. In contrast, CT images of the model group showed severe bone erosion in the paw and ankle joints, with defective joint structures, prominent osteophytes, destruction, and uneven bone surfaces. Bone destruction in the foot and ankle joints was also observed in the PB and MACP siNC groups, although the MACP siNC group exhibited a lower degree of bone destruction than the PB group. The CT images of the MACP siTNF‐α group resembled those of the healthy group, showing smooth bone surfaces and intact joint structures, without any noticeable signs of bone damage such as bone erosion or osteophytes. Bone volume fraction (BV/TV) analysis (Figure 5i) further confirmed these findings, showing a significantly lower bone volume fraction in the model group compared to the healthy group. The bone volume fraction in the MACP siTNF‐α group, however, was similar to that of the healthy group. The MACP siTNF‐α group significantly increased the BV/TV values in the articular region compared to the PB group (*p < 0.05) and the MACP siNC group (*p < 0.05).

At the end of treatment, serum was collected following euthanasia, and levels of representative pro‐inflammatory factors in the serum were measured using ELISA. Quantitative results for TNF‐α, IL‐1β, and IL‐6 are shown in Figure 5j–l, respectively. Compared to the model group, the MACP siTNF‐α group demonstrated significant reductions in serum levels of TNF‐α (**p < 0.01), IL‐1β (****p < 0.0001), and IL‐6 (****p < 0.0001), approaching the levels observed in the healthy group. Serum levels of TNF‐α (ns), IL‐1β (*p < 0.05), and IL‐6 (ns) were slightly lower in the MACP siTNF‐α group compared to the MACP siNC group.

**Figure 6:**
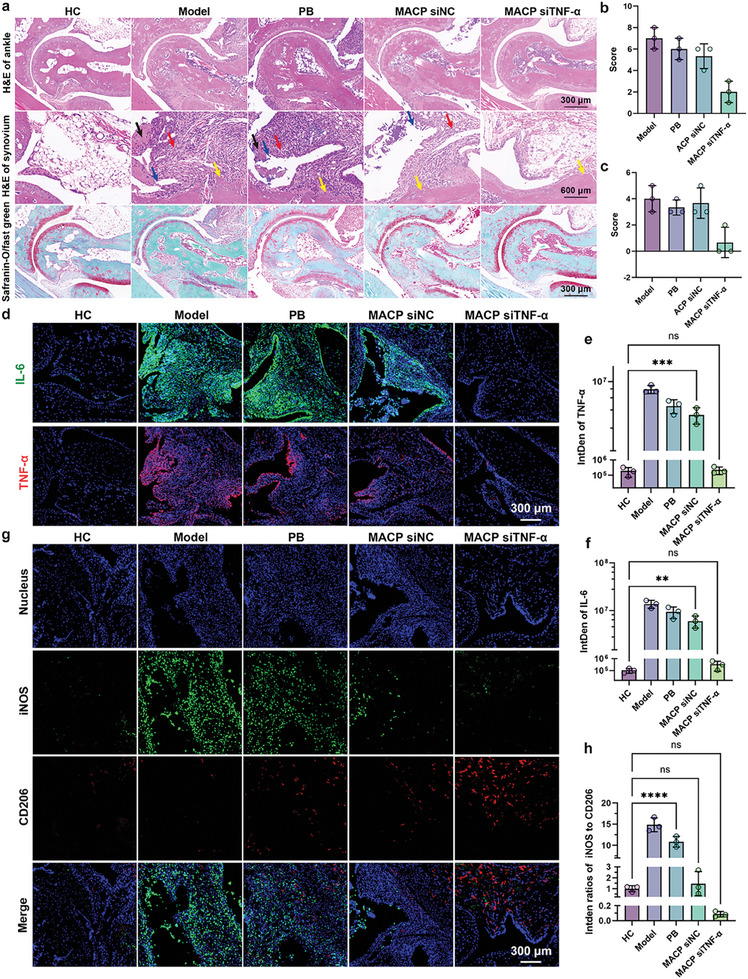
Pathological analysis of joint cavities and inflammatory microenvironment changes. a) Cartilage destruction and synovial inflammation as assessed by H&E and Safranin‐O staining. Arrows indicate synovial hyperplasia (blank), inflammatory infiltration (red), synovial cell shedding (blue), and bone erosion (yellow), *n* = 3. b) Histological synovitis score (HSS) and c) Safranin‐O/Fast Green staining score based on levels of inflammation, synovial hyperplasia, and bone or cartilage erosion, *n* = 3. d–f) Representative images and quantitative analysis of immunofluorescence staining for IL‐6 and TNF‐α in ankle joints, *n* = 3. g) Representative images of immunofluorescence staining of iNOS (M1‐type macrophages) and CD206 (M2‐type macrophages) in ankle joints following different treatments, *n* = 3. h) Fluorescence intensity ratio of iNOS to CD206, *n* = 3. Data are presented as mean ± SD, with comparisons in (e, f, h) conducted by one‐way ANOVA. *p < 0.05, **p < 0.01, ***p < 0.001, ****p < 0.0001.

Next, ankle joints were prepared as paraffin sections for hematoxylin and eosin (H&E) and Safranin‐O/Fast Green (SOFG) staining (**Figure** **6**a). H&E staining results indicated that ankle joints in the healthy group were structurally intact with smooth surfaces, loose synovial connective tissue, and well‐organized chondrocytes. In contrast, the model group showed marked inflammation, with inflammatory cell infiltration, synovial hyperplasia, connective tissue proliferation, synovial cell detachment, pannus formation, and cartilage/bone erosion. The PB group exhibited a pathological state similar to the model group, showing no significant therapeutic effect. The MACP siNC group showed some attenuation of inflammation, but inflammatory cell infiltration, pannus formation, and signs of bone destruction were still present. The MACP siTNF‐α group, however, exhibited remarkable therapeutic effects, with no significant symptoms of rheumatoid arthritis observed in the ankle joints. Quantitative analysis (Figure 6b) of the pathological features from H&E staining, using histopathological scores of the synovium (HSS),^[^ ^37^, ^38^ ^]^ revealed that the model group had the highest HSS score (7), indicating a pronounced inflammatory state. In contrast, the MACP siTNF‐α‐treated group had the lowest HSS score (2), reflecting a healthier synovial state and intact tissue structure.

The progression of rheumatoid arthritis results in the loss of cartilage protein glycogen and structural changes in collagen fiber arrangement. The cartilage (red) and osteogenic (green) conditions at the ankle joint were observed (Figure 6a) and quantitatively analyzed by SOFG staining (Figure 6c).^[^ ^39^ ^]^ SOFG staining results showed that articular cartilage in the ankle joints of mice in the healthy group was structurally intact, with well‐organized cells. In the model group, extensive loss of cartilage protein glycogen, a reduced number of chondrocytes, and lighter staining of the cartilage matrix were observed. The PB and MACP siNC groups displayed varying degrees of chondrocyte loss and collagen degradation. However, the articular cartilage in the MACP siTNF‐α group remained in a normal physiological state, indicating a significant protective effect on articular cartilage.

### 2.11 In Vivo Immunoassay

In RA, the inflammatory process is predominantly mediated and sustained by M1 macrophages, which are found both in peripheral blood and synovial tissue.^[^ ^40^ ^]^ M1/Th1 activation occurs in an inflammatory environment dominated by Toll‐like receptor (TLR) and interferon (IFN) signaling, resulting in the overproduction of pro‐inflammatory cytokines. This excessive inflammatory response contributes to osteoclastogenesis, bone erosion, and progressive joint destruction.^[^ ^25^ ^]^ Recent advances in clinical practice suggest that therapies aimed not only at suppressing pro‐inflammatory cytokines but also at promoting the polarization of M1 macrophages into anti‐inflammatory M2 macrophages could offer a more effective strategy for controlling the aberrant immune response in RA.^[^ ^41^ ^]^

The expression levels of IL‐6 and TNF‐α in the ankle region at the end of treatment were visualized and semi‐quantitatively analyzed using immunofluorescence (IF) staining (Figure 6d–f). In the model group, IL‐6 and TNF‐α levels increased by 130.22‐fold and 41.78‐fold, respectively, compared to the healthy group, indicating elevated inflammation. IL‐6 and TNF‐α levels in the PB group were similar to those in the model group, showing no significant therapeutic effect in reducing proinflammatory factors. Although proinflammatory factors were reduced in the MACP siNC group, the tissue remained in an inflamed state. The MACP siTNF‐α group demonstrated a strong anti‐inflammatory effect, reducing IL‐6 and TNF‐α levels by 94.25% and 94.35%, respectively, to levels comparable with those in the healthy group.

The targeted accumulation of MACP siTNF‐α NPs in inflammatory regions was facilitated by the preferential uptake of nanoparticles by M1‐type macrophages. This specificity is attributed to a highly efficient phagocytic uptake mechanism mediated by the interaction between anionic phospholipids on the surface of the biomimetic nanoparticles and scavenger receptor class B type 1 (SR‐B1) expressed on M1 macrophages.^[^ ^42^ ^]^ This targeted uptake enhances the delivery system's capacity to modulate macrophage polarization and improve therapeutic efficacy in the inflammatory region. To further investigate the therapeutic mechanism of MACP siTNF‐α, the distribution of M1 and M2 macrophages in the treated area was assessed by labeling iNOS (M1 macrophages) and CD206 (M2 macrophages) (Figure 6g,h). In the healthy group, both M1 and M2 macrophages were minimally present in the ankle region, with immune cells in a resting state. The model group exhibited an enrichment of M1 macrophages, with a fluorescence intensity ratio of iNOS to CD206 that was 14.84 times higher than in the healthy group. In the PB and MACP siNC groups, M1 macrophages were less enriched while M2 macrophages were more abundant. The MACP siTNF‐α group showed a significant shift from M1 to M2 macrophages, with M2 macrophages widely enriched in the observed area and a 99.42% reduction in the iNOS to CD206 fluorescence intensity ratio compared to the model group.

These results demonstrated that while intravenous injection of PB nanoenzymes modestly reduced serum inflammation levels, they failed to deliver effective therapeutic outcomes in the targeted treatment area due to their lack of specific targeting to the inflammatory regions characteristic of rheumatoid arthritis. MACP siNC improved the targeted accumulation of PB in the treatment area through biomimetic modification with macrophage membranes, effectively reducing ankle inflammation by decreasing ROS levels. However, its therapeutic efficiency was limited and inadequate in preventing inflammatory infiltration into the joint cavity. In contrast, MACP siTNF‐α demonstrated superior efficacy, achieving efficient and sustained accumulation in the inflammatory region. The MACP siTNF‐α NPs physically adsorbed ROS on their surface and catalyzed their decomposition, thereby effectively lowering ROS levels and alleviating GSH depletion within the inflammatory microenvironment. Disulfide bond‐containing guanidinium‐based polymers were rapidly degraded by GSH reduction, facilitating siRNA release to silence TNF‐α expression in the upstream inflammatory pathway. Simultaneously, PB nanoenzymes, functioning as highly effective ROS scavengers, were fully exposed intracellularly, further enhancing ROS scavenging, sustaining GSH restoration, and accelerating guanidinium polymer degradation. This innovative system formed a positive feedback loop in which the biomimetic nanoplatform reduced ROS levels, prevented GSH depletion, facilitated GSH‐mediated polymer degradation to release siRNA and expose PB nanoenzymes, and ultimately restored GSH levels while scavenging ROS. Compared to traditional gene therapy or nanoenzyme‐based methods, this dual‐function feedback mechanism synergistically improved the inflammatory microenvironment, enhanced therapeutic efficacy, and provided a robust platform for the precise treatment of rheumatoid arthritis. A reduction in reactive oxygen species (ROS) levels within M1‐type macrophages, coupled with the restoration of glutathione (GSH) levels and the inhibition of inflammatory factor expression, may facilitate the reversal of macrophage polarization.^[^ ^43^ ^]^ This process could enhance the inflammatory microenvironment, mitigate the progression of rheumatoid arthritis (RA), and promote the repair of joint tissues.^[^ ^44^ ^]^

Biofilm‐mediated biomimetic nanodrug delivery systems have emerged as a prominent area of research within the life sciences, demonstrating significant advantages in vivo drug delivery, bioimaging, and disease treatment.^[^ ^45^ ^]^ This innovative biomimetic platform employs bio‐nanotechnology to encapsulate synthetic nanoparticles within a biomimetic membrane, integrating the low immunogenicity, low toxicity, high targeting capability, and excellent biocompatibility of the biofilm with the tunability and multifunctionality of the nanocarrier.^[^ ^45^, ^46^ ^]^ As a result, it holds considerable potential for applications in precision therapy.

Compared to conventional nanodelivery systems such as polymeric micelles, liposomes, and dendrimers, the biomimetic nanoplatform used in this study provides enhanced targeting ability and prolonged circulation time, facilitated by macrophage membrane coating. This feature allows the platform to home in on inflammatory regions, thereby reducing off‐target effects and enhancing therapeutic efficiency.^[^ ^47^, ^48^, ^49^ ^]^ Furthermore, the incorporation of a positive feedback mechanism amplifies the therapeutic effect by simultaneously scavenging ROS and restoring intracellular GSH levels. However, the biomimetic platform also has certain limitations. The preparation process is more complex than traditional systems, requiring the isolation and functionalization of biological membranes, which can increase production costs and complicate scalability. Additionally, while the biofilm coating improves biocompatibility, it may pose challenges in standardizing nanoparticle characteristics, such as size and surface charge. Despite these challenges, the unique dual‐functionality and precision targeting capabilities of this platform present a promising approach for treating complex inflammatory conditions like rheumatoid arthritis.

MACP siTNF‐α nanoparticles effectively target the inflammatory microenvironment and establish a self‐sustaining positive feedback mechanism. This mechanism enhances the ROS scavenging capacity of PB nanoenzymes while improving the silencing efficiency of siRNAs on TNF‐α expression. By addressing both upstream inflammatory pathways and oxidative stress, these nanoparticles synergistically and comprehensively ameliorate the inflammatory microenvironment within the joint cavity. This dual‐mechanism approach facilitates the reversal of macrophage polarization phenotypes, promoting tissue repair and reducing inflammation‐induced damage. Clinically, this strategy has the potential to translate into improved symptom relief, delayed disease progression, and enhanced joint preservation in RA. Furthermore, the delivery system's adaptability positions it as a promising candidate for therapeutic intervention in other autoimmune and inflammatory disorders.

### 2.12 Safety Analyses

**Figure 7:**
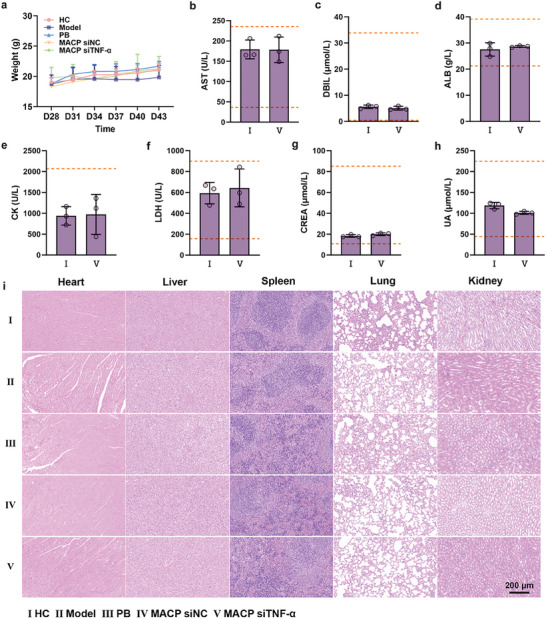
Therapeutic safety evaluation in vivo. a) Body weight changes of mice during treatment, *n* = 6. b–h) Serum collected from mice for biochemical analyses at the endpoint of treatment, *n* = 3. Blood biochemistry including aspartate aminotransferase (AST), direct bilirubin (DBIL), albumin (ALB), creatine kinase (CK), lactic dehydrogenase (LDH), creatinine (CREA) and uric acid (UA). i) Representative images of HE staining of vital organs (heart, liver, spleen, lung, and kidney) of mice after treatment, *n* = 3. Data are shown as means ± SD.

Body weight changes in each group of mice were monitored throughout the treatment period (**Figure** **7**a). Body weight trends were similar in the healthy, PB, MACP siNC, and MACP siTNF‐α groups, with mice showing gradual weight gain. Mice in the model group exhibited the slowest rate of weight gain, likely due to the high inflammatory state, but did not experience weight loss. At the end of the treatment period, serum was collected, and no significant differences were found between the MACP siTNF‐α and healthy groups regarding blood biochemical indicators (Figure 7b–h), with all biochemical levels within normal physiological ranges. Major organs (heart, liver, spleen, lungs, and kidneys) from each group were also collected and examined via H&E staining to assess the physiological impact of the treatment (Figure 7i). No significant pathological changes were observed. These results demonstrated that MACP siTNF‐α exhibited good biocompatibility, and the treatment regimen was safe, supporting its potential as a safe and effective multifunctional delivery system for targeted therapy of rheumatoid arthritis.

Bionic membranes, as sophisticated carriers derived from biological systems, face several technical and practical hurdles when advancing from laboratory research to clinical application.^[^ ^50^ ^]^ Key challenges include stability, scalability, and the unclear biological effects of biomimetic nanodelivery systems.^[^ ^51^ ^]^ Active surface substances on biomimetic membranes are prone to inactivation, posing significant issues for long‐term storage and stability.^[^ ^52^ ^]^ Additionally, current manufacturing technologies are underdeveloped, with challenges including inefficient isolation and purification processes, incomplete surface modifications, and limited loading and delivery efficiencies.^[^ ^53^ ^]^ The complex preparation protocols further complicate large‐scale production, presenting a barrier to clinical translation.^[^ ^54^ ^]^ Moreover, the biological effects of biomimetic delivery systems in human patients remain inadequately understood. Extensive in vivo research is crucial to comprehensively evaluate potential side effects and therapeutic efficacy. Safety concerns, such as potential immunogenicity or off‐target effects, must be thoroughly addressed to ensure clinical applicability.^[^ ^55^ ^]^ To overcome these limitations, future research should focus on refining scalable manufacturing processes, developing robust stabilization techniques, and elucidating the long‐term biological effects of biomimetic systems. Investigating the interactions between biomimetic carriers and human immune systems will also be pivotal for optimizing their design and enhancing their safety profile. This groundwork is essential for translating these advanced delivery systems into clinical applications and ensuring their efficacy and safety in treating diseases such as rheumatoid arthritis.

## 3 Conclusion

In conclusion, this study introduced a co‐delivery system combining nanoenzymes and gene therapeutics, sequentially modified with guanidinium‐based polymers and macrophage membranes to achieve synergistic therapeutic effects. MACP siTNF‐α NPs efficiently targeted the inflammatory site, creating a biomimetic nanodelivery platform capable of constructing a positive feedback mechanism within inflamed tissues. This system facilitated the optimal exposure of PB nanoenzymes for effective ROS scavenging and the rapid release of siRNAs to silence TNF‐α expression in the upstream inflammatory pathway. Through its dual mechanism, MACP siTNF‐α NPs significantly improved the inflammatory microenvironment in the joint cavity, promoted macrophage polarization reversal, and achieved effective treatment of RA. Furthermore, the delivery system shows great potential which could be seamlessly extended to other inflammatory diseases of the autoimmune system.

## 4 Experimental Section

### Materials, Cell Lines, and Animals

All chemicals were purchased from commercial suppliers and used without further purification. Povidone K30 (PVP K30) was purchased from BASF SE (Ludwigshafen, Germany). Potassium ferricyanide (K_3_[Fe(CN)_6_]), sodium ethylenediaminetetraacetic acid 2 (EDTA‐2Na), and agmatine sulfate (AGM) were sourced from Shanghai Yuanye Bio‐Technology Co., Ltd. (Shanghai, China). RNAiso plus, TB green real time PCR kit (TKR‐RR820A), and gDNA eraser kit (TKR‐RR047A) were purchased from Takara Biotechnology (Japan). Glutathione (GSH), l‐glutathione, green lysosomal probe, hoechst 33342, Coomassie brilliant blue, diethyl pyrocarbonate, BCA kit (P0010), catalase assay kit (S0051), total SOD activity detection kit (S0101S), and nitric oxide assay kit (S0021S) were obtained from Biyuntian BioTECH Co. Ltd. (Shanghai, China). Tris, glycylglycine, chlorpromazine, amiloride, genistein, β‐cyclosin, block lipid transport‐1 (BLT‐1), and DCFH‐diacetate (DCFH‐DA) were purchased from Solaibao (Beijing, China). Fetal bovine serum was purchased from Lonsera (Shanghai, China). Dulbecco's modified eagle's medium (DMEM) and RPMI‐1640 medium were obtained from Corning Cellgro Inc (Herndon, VA, USA). Horseradish enzyme labeling goat anti‐rabbit IgG was sourced from Zhongshan JinQiao Shanghai Co., Ltd. (Shanghai, China). Rabbit anti‐beta‐actin polyclonal antibody, 4′,6‐diamidino‐2‐phenylindole (DAPI), polyvinylidene fluoride membrane (PVDF), phosphate‐buffered saline (PBS), rabbit anti‐mouse IL‐6 antibody, rabbit anti‐mouse iNOS antibody, and rabbit anti‐mouse CD206 antibody were purchased from Wuhan Saiweier Biotechnology Co., Ltd. (Wuhan, China). Agarose, *N*,*N'*‐cystamine‐bis‐acrylamide (CBA), and lipopolysaccharide (LPS) were obtained from Sigma‐Aldrich (Shanghai, China). 1,1‐Dioctadecyl‐3,3,3,3‐tetramethylindotricarbocyaine iodide (DiR) was purchased from Meilunbio (Dalian, China). Mouse TNF‐α ELISA kit (EMC102a, Xinbosheng), mouse IL‐6 ELISA Kit (EMC004QT), and mouse IL‐1β ELISA Kit (EMC001b) were obtained from were obtained from NeoBioscience (Shenzhen, China). BV421 rat anti‐mouse CD86 Antibody and PE rat anti‐mouse CD206 antibody were purchased from BD (Franklin Lakes, NJ, USA). Bovine type II collagen and complete Freund's adjuvant were obtained from Chondrex, Inc. (Redmond, WA, USA). Rat anti‐mouse TNF alpha antibody was purchased from Abcam (Cambridge, MA, USA). siTNF‐α sequences were synthesized by Sangon Biotech Co., Ltd. (Shanghai, China), and the sequences were provided in the supporting materials Table S1 (Supporting Information). The mouse mononuclear macrophage (RAW 264.7) was obtained from the Wuhan Servicebio Biotechnology Co., Ltd. (Wuhan, China). Mouse mononuclear macrophages (RAW 264.7) were obtained from Wuhan Servicebio Biotechnology Co., Ltd. (Wuhan, China) and cultured in DMEM medium at 37 °C in a humidified 5% CO_2_ incubator following standard protocols. Eight‐week‐old male DBA/1JGpt mice were acquired from the GemPharmatech Co., Ltd. (production license SCXK [Su] 2023‐0009, Nanjing, China). The animal experiments were conducted according to the protocols of the National Regulation of China for Care and Use of Laboratory Animals and were simultaneously approved by the Ethical Committee of Shenzhen Technology University (SZTUDWLL2024009).

### Synthesis and Characterization of AC

Low molecular weight guanidinium‐based polymers containing disulfide bonds (designated as AC) were synthesized through a Michael addition polymerization reaction.^[^ ^20^ ^]^ Equimolar amounts of *N*,*N'*‐cystamine‐bis‐acrylamide (CBA) and agmatine sulfate (AGM) were used as substrates, with 50% methanol (v/v) serving as the reaction medium. The pH of the system was adjusted to 11 with lithium hydroxide, and the reaction was carried out at 50 °C for 7 days under nitrogen protection and light‐avoiding conditions. Upon completion, the product was diluted with deionized water, and the pH was adjusted to 4 using HCl to enhance its water solubility. The product was then purified by dialysis and freeze‐dried to obtain AC. For characterization, the polymer was dissolved in deuterated water and analyzed using hydrogen nuclear magnetic resonance (^1^H‐NMR) spectroscopy at 400 MHz (AVANCE NEO 400, Bruker Switzerland AG). Additionally, the polymer was dissolved in deionized water and subjected to matrix‐assisted laser desorption ionization‐time‐of‐flight mass spectrometry (MALDI‐TOF/MS) using an UltrafleXtreme (Bruker, Germany) with α‐cyano‐4‐hydroxycinnamic acid (CHCA) as the matrix. To assess the binding and encapsulation efficiency of AC on siRNA, AC was co‐incubated with siRNA at various mass ratios (1:1, 2:1, 3:1, 4:1, 5:1) for 30 min, and agarose gel electrophoresis was performed.

### Preparation of Prussian Blue Nanoenzymes

Prussian blue (PB) nanoenzymes were synthesized via a hydrothermal method as previously reported.^[^ ^56^ ^]^ PVP‐K30 (5 g) was dissolved in HCl (40 mL, 1 mol L^−1^) under magnetic stirring at 1200 rpm. Potassium ferricyanide (396 mg) was then added, and stirring continued until complete dissolution, resulting in a ferric yellow solution. The reaction was conducted at 80 °C with magnetic stirring at 860 rpm for 20 h. Upon completion, the solution turned blue. After allowing the mixture to cool to room temperature, acetone (120 mL) was added, and the mixture was centrifuged at 12 000 rpm for 10 min at 4 °C. The supernatant was discarded, and the blue precipitate was washed twice with ethanol. The precipitate was then vacuum‐dried at 50 °C overnight to yield PB nanoparticles, which were collected, weighed, and stored in an airtight container. Hydrogen peroxidase and superoxide dismutase (SOD) activities of PB nanoparticles were measured using standard assay kits according to the manufacturer's instructions.

### Macrophage Membrane Preparation

Murine macrophages (RAW 264.7) were cultured in DMEM complete medium containing 10% fetal bovine serum and 1% penicillin‐streptomycin. When cell density exceeded 90%, the medium was discarded, and cells were washed with cold PBS. Cells were resuspended in PBS and collected by centrifugation at 1400 rpm for 3 min. The cell pellet was resuspended in PBS (1 mM phosphate, containing 1 mM PMSF), followed by sonication for 10 min (25 W, 3 s ON / 5 s OFF) on ice using a probe sonicator (JY92‐IIN, Xinyi, China). After sonication, the suspension was centrifuged at 2800 rpm for 10 min, and the supernatant was collected. This supernatant was further centrifuged at 16 000 rpm for 20 min, after which the supernatant was discarded, leaving the macrophage membrane (MCM) as the precipitate. MCM was resuspended in PBS, and the membrane protein concentration was determined using a BCA protein assay (Biyuntian, China). Phospholipid membrane mass was then extrapolated, and the MCM was stored in liquid nitrogen for long‐term preservation.

### Preparation of MACP siTNF‐α NPs

To prepare MACP siTNF‐α nanoparticles, a mixture of siTNF‐α and AC polymer (w/w, 1:100) was first co‐incubated under magnetic stirring for 30 min. Subsequently, PB (siTNF‐α: PB, 1:20, w/w) was added, and the solution was stirred for an additional 30 min to form ACP siTNF‐α NPs. For biomimetic modification, macrophage membrane (equivalent to 2.5 times the mass of the inner core) was added to the co‐incubation system and stirred for 20 min to ensure thorough mixing. The system was then sonicated for 10 min (25 W, 3 s ON/5 s OFF) in an ice bath using a probe‐based ultrasound device. Following sonication, the mixture was centrifuged at 16 000 rpm at 4 °C for 20 min. The precipitate was collected and resuspended in PBS or normal saline (NS), resulting in an aqueous dispersion of MACP siTNF‐α NPs. siNC was a negative control sequence for siRNA, serving as a nonspecific RNA sequence to demonstrate the specificity of siTNF‐α silencing. The delivery system for drug‐loaded siNC was defined as MACP siNC.

### Characterization of Formulations

Appropriate amounts of PB NPs, ACP siTNF‐α NPs, and MACP siTNF‐α NPs were each dispersed in deionized water. Particle size distributions, polydispersity indices (PDI), and surface charges of the nanoparticles were determined using dynamic light scattering (DLS) (ZS XPLORER, Malvern Panalytical Ltd., Malvern, UK). The colloidal stability of MACP siTNF‐α NPs was assessed over 6 days. For microscopic analysis, the deionized aqueous dispersion of nanoparticles was applied dropwise onto a copper mesh, air‐dried, and the microscopic morphology and monodispersity of PB NPs, ACP siTNF‐α NPs, and MACP siTNF‐α NPs were observed using a transmission electron microscope (TEM) (Talos F200X G2, Thermo Scientific, USA). Elemental distributions were analyzed through mapping scans. To determine the encapsulation efficiency (EE%) and drug loading rate (DL%) of siRNA, MACP siTNF‐α NPs were prepared with siTNF‐α modified by FAM (Ex: 494 nm, Em: 530 nm). The supernatant from the preparation process was collected, and the concentration of free siTNF‐α was measured using a multifunctional microplate reader (Synergy H1, BioTek, Winooski, VT, USA). Encapsulation efficiency (EE%) and drug loading rate (DL%) of siRNA were calculated by formula (1) and formula (2). Formula (1): EE% = (content of initially added drug – content of free drug in the supernatant)/content of initially added drug × 100. Formula (2): DL% = (amount of initially added drug – amount of free drug in the supernatant)/amount of drug‐loaded nanoparticles × 100. Agarose gel electrophoresis was used to evaluate the encapsulation ability of the preparations for siRNA. Briefly, AC siTNF‐α, ACP siTNF‐α NPs, and MACP siTNF‐α NPs (AC: siRNA = 100:1, w/w) were mixed with a loading buffer and subjected to agarose gel electrophoresis, then imaged using a gel imaging system (iBright 1500, Thermo, USA). To confirm membrane protein transfer to the surface of MACP siTNF‐α NPs during the MCM membrane coating process, SDS‐PAGE analysis was performed. Equal amounts of proteins were loaded onto 12% polyacrylamide gels, electrophoresed at 120 V for 40 min, stained with Coomassie Blue, and photographed for documentation.

### In Vitro siRNA Release

The release mechanism of siRNA was examined using Cy5‐labeled siRNA through dialysis in different media: pH 7.4 PBS, pH 7.4 PBS containing 1 mM GSH, pH 5.5 PBS, and pH 5.5 PBS containing 1 mM GSH. Briefly, 1 mL of ACP siTNF‐α NPs or MACP siTNF‐α NPs (siRNA concentration of 4.67 µg mL^−1^) was placed in a dialysis bag (MW: 100 kDa), and the release experiments were conducted at 37 °C with 100 rpm oscillation. At predetermined time points (0, 0.5, 1, 2, 4, 6, 8, 12, 24, and 48 h), 100 µL samples were collected and immediately replenished with an equal volume of fresh dialysis medium. Each group included three parallel samples. The free siRNA content was determined using a multifunctional microplate reader, and the cumulative release rate of siRNA was calculated and plotted to create a release curve.

### Serum Stability

Serum stability assays were conducted to preliminarily evaluate the interaction of MACP siTNF‐α NPs with serum proteins. Briefly, 100 µL of MACP siTNF‐α NPs at various concentrations (equivalent PB concentrations of 20, 40, 80, 100, and 160 µg mL^−1^) were mixed with 100 µL of fetal bovine serum (FBS) in 96‐well plates and incubated statically at 37 °C. The final FBS concentration was 50% (v/v), with a 50% FBS group used as a blank control. After static incubation for 0, 1, 4, 8, 12, and 24 h, absorbance was measured at 560 nm (A560). Five samples were measured in parallel for each time point, and the change in absorbance was calculated using the following formula (3): Change in absorbance (a.u.) = *A*_experimental group_ − *A*_blank control group_.

### Hemolysis Rate

The biocompatibility of ACP siTNF‐α NPs and MACP siTNF‐α NPs with red blood cells was evaluated by measuring the hemolysis rate. Fresh mouse blood was washed with PBS, centrifuged to collect erythrocytes, and resuspended in normal saline (NS) to prepare a 4% (v/v) erythrocyte suspension. For each test, 250 µL of erythrocyte suspension was mixed with 250 µL of deionized water (DI), NS, or various concentrations of ACP siTNF‐α NPs or MACP siTNF‐α NPs in EP tubes. Final nanoparticle concentrations were equivalent to siRNA concentrations of 0.1, 1, 10, 50, 100, and 200 nM. The mixtures were incubated at 37 °C for 6 h, followed by centrifugation at 720 × *g*g at 4 °C for 10 min. The appearance of each sample was photographed, and the supernatants were transferred to 96‐well plates for absorbance measurement at 540 nm (A540). Each group included three parallel samples. The hemolysis rate was calculated using the following formula (4): Hemolysis ratio (%) = (*A*_preparation_ − *A*_NS_)/(*A*_DI_ − *A*_NS_) × 100.

### Lipopolysaccharide‐Induced Macrophage Polarization

Macrophages, as immune cells, can polarize into different phenotypes in response to varying physiological and pathological environments both in vivo and ex vivo. To induce M1‐type polarization, macrophages were co‐incubated with lipopolysaccharide (LPS, 1 µg mL^−1^), establishing an inflammatory cell model. The effectiveness of the induction was verified by measuring the nitric oxide (NO) levels in the cell culture supernatant using a Griess reagent assay, following the kit instructions.

### Cytotoxicity

RAW 264.7 cells were seeded at a density of 5 × 10^3^ cells per well in clear 96‐well plates and incubated overnight. Cells without LPS treatment were designated as M0‐type macrophages, while those stimulated with LPS were classified as M1‐type macrophages. Different concentrations of ACP siTNF‐α NPs or MACP siTNF‐α NPs (100 µL, corresponding to siRNA concentrations of 0.1, 1, 10, 50, 100, and 200 nM) were added to the wells. The culture medium alone served as a blank group, and untreated cells were used as a control group. After 24 h of incubation at 37 °C, 10 µL of CCK‐8 solution was added to each well, followed by an additional 1‐h incubation. Absorbance was measured at 450 nm using a multifunctional microplate reader, and cell viability was calculated by the formula (5): Cell viability (%) = (*A*_experimental group_ − *A*_blank group_)/(*A*_control group_ − *A*_blank group_) × 100.

### Cellular Uptake Evaluation

To assess cellular uptake, Cy5‐labeled siRNA was used to evaluate the uptake of agents by macrophages in different polarization states using flow cytometry (FACSymphony A1, BD Biosciences, San Diego, CA) and confocal laser scanning microscopy (CLSM, STELLARIS 5, Leica, Germany). RAW 264.7 cells were seeded in 6‐well plates at a density of 5 × 10⁵ cells per well and incubated overnight. Cells were then either stimulated with LPS (model group) or left unstimulated (blank group). Free siRNA, ACP siTNF‐α NPs, and MACP siTNF‐α NPs were co‐incubated with cells for 4 h at an equal Cy5‐siRNA concentration of 50 nM. After co‐incubation, cells were washed with PBS and analyzed by flow cytometry. The concentration‐dependent uptake of MACP siTNF‐α NPs by M1‐type macrophages was also investigated using the same co‐incubation protocol. Additionally, cellular uptake was visualized using CLSM. After co‐incubation, cells were fixed with 4% paraformaldehyde, and nuclei were stained with Hoechst 33 342 (Ex: 350 nm, Em: 461 nm). Samples were then imaged using CLSM.

### Mechanisms of Cellular Uptake

The internalization mechanism of MACP siTNF‐α NPs by M1‐type macrophages was investigated by introducing various uptake inhibitors into the co‐incubation system. RAW 264.7 cells were seeded at a density of 5 × 10⁵ cells per well in 6‐well plates and incubated overnight. Cells were stimulated with LPS prior to testing. To examine specific uptake pathways, cells were pretreated with the following inhibitors for 1 hour: chlorpromazine (20 µM, clathrin‐mediated endocytosis), amiloride (30 µM, macropinocytosis), genistein (100 µM, caveolin‐mediated endocytosis), β‐cyclodextrin (3 mM, lipid raft‐mediated endocytosis), and BLT‐1 (1 mM, scavenger receptor‐mediated phagocytosis). After pretreatment, the medium was replaced with fresh complete medium containing MACP siTNF‐α NPs (equivalent to Cy5‐siRNA at 50 nM) along with the respective inhibitors, and co‐incubation was continued for 6 h. Additionally, a separate group of cells was co‐incubated at 4 °C to assess the energy dependence of cellular uptake. At the end of the co‐incubation period, cells were collected and analyzed by flow cytometry. The uptake inhibition rate (%) was calculated using the following equation (6): Uptake inhibition rate (%) = (mean fluorescence intensity of control group – mean fluorescence intensity of inhibitor group)/mean fluorescence intensity of control group × 100.

### Lysosomal Escape

RAW 264.7 cells were seeded at a density of 5 × 10⁵ cells in confocal dishes and cultured overnight, with LPS treatment to induce polarization to the M1 type. Cy5‐labeled MACP siTNF‐α was then added and co‐incubated with the cells for either 1 h or 3 h. At the end of the co‐incubation period, cells were washed with PBS, and nuclei were stained with Hoechst 33342. Lysosomes were stained with LysoTracker Green (Ex: 504 nm, Em: 511 nm). The lysosomal escape efficiency of the preparations was then visualized using confocal laser scanning microscopy (CLSM).

### Nanoenzymatic Activity

To assess catalase (CAT) activity, gradient concentrations of PB NPs (20, 40, 80, 100, and 160 µg mL^−1^) as well as ACP siTNF‐α NPs and MACP siTNF‐α NPs (equivalent PB concentration of 100 µg mL^−1^) were added to a catalase assay buffer (150 mM) and incubated at room temperature for 6 h. The reaction was terminated, and color development was performed according to the manufacturer's instructions. CAT activity was evaluated by measuring absorbance at 520 nm (A520). To analyze superoxide scavenging activity, the WST‐8 method was used with a total SOD activity assay kit, following the manufacturer's instructions. Gradient concentrations of PB NPs (20, 40, 80, 100, and 160 µg mL^−1^) as well as ACP siTNF‐α NPs and MACP siTNF‐α NPs (equivalent PB concentration of 100 µg mL^−1^) were mixed with the WST‐8/enzyme working solution and incubated at 37 °C for 30 min. Absorbance was then measured at 450 nm to calculate SOD activity.

### Evaluation of Intracellular GSH Levels

RAW 264.7 cells were seeded at a density of 5 × 10⁵ cells per well in 6‐well plates and incubated overnight. Cells were stimulated with LPS (+LPS group) or left untreated (–LPS group). Gradient concentrations of PB NPs (20, 30, 40, 160 µg mL^−1^) as well as ACP siTNF‐α NPs and MACP siTNF‐α NPs (equivalent PB concentration of 30 µg mL^−1^) were co‐incubated with the cells for 12 h. Afterward, cells were either re‐stimulated with LPS or left untreated for an additional 4 h. Cells were then washed with PBS, and collected by centrifugation, and the cell pellet was resuspended with a protein removal reagent. Cell lysis was achieved through repeated freeze‐thaw cycles, followed by centrifugation at 10 000 × *g* for 5 min at 4 °C to collect the supernatant. The supernatant was incubated with a total glutathione assay working solution for 5 min according to the kit instructions (Total Glutathione Assay Kit, Beyotime, China). Next, an NADPH solution was added, and mixed, and the absorbance at 412 nm was measured after a 25‐min reaction to calculate intracellular total glutathione content.

### Evaluation of ROS Scavenging Capacity

RAW 264.7 cells were seeded at a density of 2 × 10⁵ cells per well in 12‐well plates and incubated overnight. Cells were either stimulated with LPS (+LPS group) or left untreated (‐LPS group). PB NPs, ACP siTNF‐α NPs, and MACP siTNF‐α NPs were then co‐incubated with the cells for 24 h, with each group adjusted to a PB concentration of 100 µg mL^−1^. Following co‐incubation, cells were washed with PBS and subsequently incubated with the ROS probe 2′,7′‐dichlorofluorescein diacetate (DCFH‐DA, Ex: 488 nm, Em: 525 nm) for 30 min. After incubation, cells were thoroughly washed with basal medium, and intracellular ROS levels were visualized using an inverted fluorescence microscope (DMi8, Leica, Wetzlar, Germany).

### RT‐qPCR

RAW 264.7 cells were seeded at a density of 5 × 10⁵ cells per well in 6‐well plates and incubated overnight. MACP siNC NPs and MACP siTNF‐α NPs were then co‐incubated with the cells for 24 h, with each group adjusted to a siRNA concentration of 100 nM. After co‐incubation, LPS was added, and incubation continued for an additional 3 h. Total mRNA was extracted following standard protocols, reverse transcribed to cDNA, and analyzed by RT‐qPCR using a QuantStudio 5 system (Thermo Fisher Scientific, USA). The siRNA inhibition rate was calculated by relative quantification using the Livak method, with β‐actin as the internal reference gene. Primer sequences are provided in the supporting materials (Table S2, Supporting Information).

### Western Blotting

RAW 264.7 cells were seeded at a density of 5 × 10⁵ cells per well in 6‐well plates and incubated overnight. Cells were either stimulated with LPS (+LPS group) or left untreated (–LPS group). MACP siNC NPs and MACP siTNF‐α NPs were co‐incubated with the cells for 12 h at an siRNA concentration of 100 nM. After co‐incubation, cells were washed with PBS, and total protein was extracted using RIPA lysis buffer, and incubated for 30 min in an ice bath. The lysate was centrifuged at 13 000 rpm for 15 min, and the supernatant was collected for protein quantification using a BCA assay. Equal amounts of protein were loaded onto 4–20% polyacrylamide gels and separated by electrophoresis at 120 V. Proteins were then transferred onto PVDF membranes at 95 V for 25 min. The membranes were blocked with 5% skimmed milk at room temperature for at least 1 hour, then incubated overnight at 4 °C with primary antibodies against TNF‐α (1:1000) and β‐Actin (1:1000). After primary incubation, membranes were treated with a secondary antibody (1:1500) for 2 h at room temperature. Following thorough washing, an ultrasensitive ECL chemiluminescent reagent was applied to visualize the proteins, and images were captured using a gel imaging system.

### Enzyme‐Linked Immunosorbent Assays

RAW 264.7 cells were seeded at a density of 5 × 10⁵ cells per well in 6‐well plates and incubated overnight. MACP siNC NPs and MACP siTNF‐α NPs were then co‐incubated with the cells for 24 h at an siRNA concentration of 100 nM. After co‐incubation, LPS was added, and incubation was continued for an additional 4 h. The supernatants were collected, and proteins were concentrated using the chloroform/methanol method. Protein concentrations were determined using a BCA assay. After adjusting to equal protein concentrations, the levels of secreted TNF‐α and IL‐6 in the supernatants were measured using enzyme‐linked immunosorbent assay (ELISA).

### Examination of Macrophage Polarization Phenotypes

RAW 264.7 cells were seeded at a density of 2 × 10⁵ cells per well in 12‐well plates and incubated overnight. Cells were either stimulated with LPS (+LPS group) or left untreated (–LPS group). PB NPs, ACP siTNF‐α NPs, and MACP siTNF‐α NPs were then co‐incubated with the cells for 24 h, with each group standardized to a PB concentration of 100 µg mL^−1^. At the end of the co‐incubation, cells were collected and stained to identify macrophage polarization phenotypes. BV421 rat anti‐mouse CD86 antibody was used to label M1‐type macrophages, and PE rat anti‐mouse CD206 antibody was used to label M2‐type macrophages. Staining was performed for 30 min at 4 °C in the dark, and macrophage polarization was subsequently analyzed by flow cytometry.

### Construction of Collagen‐Induced Arthritis (CIA) Model

Eight‐week‐old male DBA/1J mice were housed under standard conditions. For the first immunization, a 100 µL emulsion of bovine type II collagen and complete Freund's adjuvant was injected. A booster immunization was administered three weeks later with a 100 µL emulsion of bovine type II collagen and incomplete Freund's adjuvant. Twenty‐eight days after the first immunization, the onset of arthritis was used as the starting point for treatment.

### Biodistribution

MACP siTNF‐α NPs were labeled with the near‐infrared fluorescent dye DiR (Ex: 748 nm, Em: 780 nm). CIA mice and healthy mice received tail vein injections of MACP siTNF‐α NPs at an siRNA‐equivalent dose of 1 mg kg^−1^ body weight. At designated time points (0, 2, 4, 6, 8, 12, 24, 36, and 48 h postinjection), mice were anesthetized with an isoflurane gas system, and the accumulation of the agent in the limbs was observed using a small animal imaging system (Vevo LAZR‐X, FUJIFILM VisualSonics, Inc., Toronto, Canada). After 48 h, the mice were euthanized, and the heart, liver, spleen, lungs, kidneys, and limbs were collected for ex vivo imaging. Fluorescence signals were analyzed using Micro‐Manager software. Each group included three mice observed in parallel.

### In Vivo Therapeutic Efficacy in CIA Mice

CIA mice were randomly divided into four groups: model group, PB group, MACP siNC group, and MACP siTNF‐α group, with six mice per group. Each mouse received tail vein injections of the drug (equivalent to siRNA 0.4 mg kg^−1^ body weight and PB 8 mg kg^−1^ body weight) once every 3 days, for a total of five doses. Healthy, untreated mice served as the control group. Every 3 days, the thickness of the hind paw and the width of the ankle were measured using digital Vernier calipers. Arthritic inflammation was assessed based on appearance, and clinical scores were recorded according to specific guidelines provided in Table S3 (Supporting Information) of the supporting materials.

### Bone Analysis by Micro‐CT

At the end of the treatment cycle, mice were euthanized, and hind limbs were collected and fixed in 4% paraformaldehyde. The bone microstructures were analyzed using microcomputed tomography (micro‐CT, NMC‐200, NEMO, China), and images were reconstructed and assessed. Three mice were analyzed in parallel for each group.

### Histological Analysis of Mouse Ankles

After fixation in 4% paraformaldehyde, ankle joints were prepared as paraffin sections. H&E staining and Safranin‐O/Fast Green staining were performed to assess pathological changes. Quantitative analysis of ankle joint pathology was conducted using the synovial histopathology score (HSS) and Safranin‐O/Fast Green staining score, as outlined in Tables S4 and S5 (Supporting Information). Each group included three mice analyzed in parallel.

### Immunoassay

At the treatment endpoint, serum was collected, and levels of inflammatory factors TNF‐α, IL‐6, and IL‐1β were measured using ELISA. Paraffin sections of mouse ankle joints were sequentially deparaffinized, subjected to antigen retrieval, and blocked with BSA. Sections were then stained with the following antibodies: rabbit anti‐mouse TNF‐α, rabbit anti‐mouse IL‐6, rabbit anti‐mouse iNOS (a marker for M1‐type macrophages), and rabbit anti‐mouse CD206 (a marker for M2‐type macrophages). Following antibody staining, cell nuclei were stained with DAPI (Ex: 340 nm, Em: 488 nm), and immunofluorescence was observed. Each group included three mice analyzed in parallel.

### Safety Evaluation

The body weights of mice in each group were monitored and recorded at three‐day intervals throughout the treatment period. At the treatment endpoint, serum was collected, and major biochemical indices, including serum alanine aminotransferase (ALT), aspartate aminotransferase (AST), creatinine (CREA), uric acid (UA), creatine kinase (CK), lactate dehydrogenase (LDH), blood urea nitrogen (BUN), and direct bilirubin (DBIL), were measured using standardized assay kits. The main organs (heart, liver, spleen, lungs, and kidneys) were collected from each group, prepared as paraffin sections, and analyzed with H&E staining. Each group included three mice analyzed in parallel.

### Statistical Analysis

Statistical analyses were conducted using GraphPad Prism 10 software, and data are presented as mean ± standard deviation (SD). A two‐tailed unpaired Student's *t*‐test was used for comparisons between two groups, while one‐way ANOVA was used for comparisons among multiple groups. Significance levels were denoted as follows: *p < 0.05, **p < 0.01, ***p < 0.001, and ****p < 0.0001.

## Conflict of Interest

The authors declare no conflict of interest.

## Supporting information



Supporting Information

## Data Availability

The data that support the findings of this study are available from the corresponding author upon reasonable request.
