# Supplementary material for: Biomimetic Nanoplatform for Targeted Rheumatoid Arthritis Therapy: Modulating Macrophage Niches Through Self‐Sustaining Positive Feedback‐Driven Drug Release Mechanisms
Source: Adv Sci (Weinh). 2025 Feb 22;12(15):2416265. doi: 10.1002/advs.202416265 (PMC12005813; doi:10.1002/advs.202416265)
Supplement: Supplementary file 1 — Supporting Information [file ADVS-12-2416265-s001.docx]

Supporting Information

Biomimetic Nanoplatform for Targeted Rheumatoid Arthritis Therapy: Modulating Macrophage Niches through Self-Sustaining Positive Feedback-Driven Drug Release Mechanisms

Huichao Xie, Xiaoyu Huang, Bao Li, Yongfeng Chen, Haoran Niu, Tong Yu, Shimei Yang, Shuxin Gao, Yutong Zeng, Tianzhi Yang, Yan Kang, Keda Zhang*, Pingtian Ding*

**Supplemental Tables**

**Table S1. Small interfering RNA (siRNA) sequences.**

| siRNA | Sense (5'-3') | Antisense (5'-3') |
| --- | --- | --- |
| TNF-α | GACAACCAACUAGUGGUGCTT | GCACCACUAGUUGGUUGUCTT |
| Negative control | UUCUCCGAACGUGUCACGUTT | ACGUGACACGUUCGGAGAATT |

**Table S2. RT-qPCR primers.**

| Gene | Forward (5'-3') | Reverse (5'-3') |
| --- | --- | --- |
| TNF-α | GACGTGGAACTGGCAGAAG | TTGGTGGTTTGTGAGTGTG |
| β-actin | GTGCTATGTTGCTCTAGACTTCG | ATGCCACAGGATTCCATACC |

**Table S3. Qualitative scoring system for assessing paw inflammation severity.**

| Score | Condition |
| --- | --- |
| 0 | Normal. |
| 1 | Mild erythema or redness mainly in the joints. |
| 2 | Mild swelling or erythema extending from the ankle to the tarsus. |
| 3 | Erythema and moderate swelling extending from the ankle to the metatarsal joints. |
| 4 | Erythema and severe swelling encompassing the ankle, foot, digits, or ankylosis. |

All four paws were scored, with a maximum clinical score of 16 per mouse.

**Table S4. Synovial histopathology score (HSS).**

| Score | Feature |
| --- | --- |
| A. Hyperplasia or enlargement of the synovial lining cell layer | |
| 0 | Absent. |
| 1 | Slight enlargement (two to three cell layers). Rare giant cells. |
| 2 | Moderate enlargement (four to five cell layers). Some giant cells or lymphocytes. |
| 3 | Strong enlargement (more than six cell layers). Frequent giant cells and lymphocytes. |
| B. Inflammatory infiltration | |
| 0 | Absent. |
| 1 | Slight infiltration (diffusely located single cells or small perivascular aggregates of lymphocytes). |
| 2 | Moderate infiltration (lymphatic aggregates or small lymphatic follicles without germinal centers). |
| 3 | Strong infiltration (lymphatic follicles with germinal centers or confluent subsynovial infiltration). |
| C. Activation of synovial stroma/pannus formation | |
| 0 | Absent. |
| 1 | Slight activation (low cellularity with slight edema or fibroblast activity). |
| 2 | Moderate activation (moderate cellularity with fibroblasts, endothelial cells, or giant cells). |
| 3 | Strong activation (high cellularity with dense fibroblasts, endothelial cells, and abundant giant cells). |

**Table S5. Safranin-O/Fast Green staining score.**

| Score | Feature |
| --- | --- |
| 0 | Normal. |
| 1 | Mild chondrocyte loss and/or collagen disruption. |
| 2 | Focal mild (superficial) chondrocyte loss and/or collagen disruption. |
| 3 | Moderate multifocal (middle-zone depth) chondrocyte loss and/or collagen disruption. |
| 4 | Significant multifocal (deep-zone depth) chondrocyte loss and/or collagen disruption. |
| 5 | Severe multifocal (to tidemark depth) chondrocyte loss and/or collagen disruption. |

**Supplemental Figure**


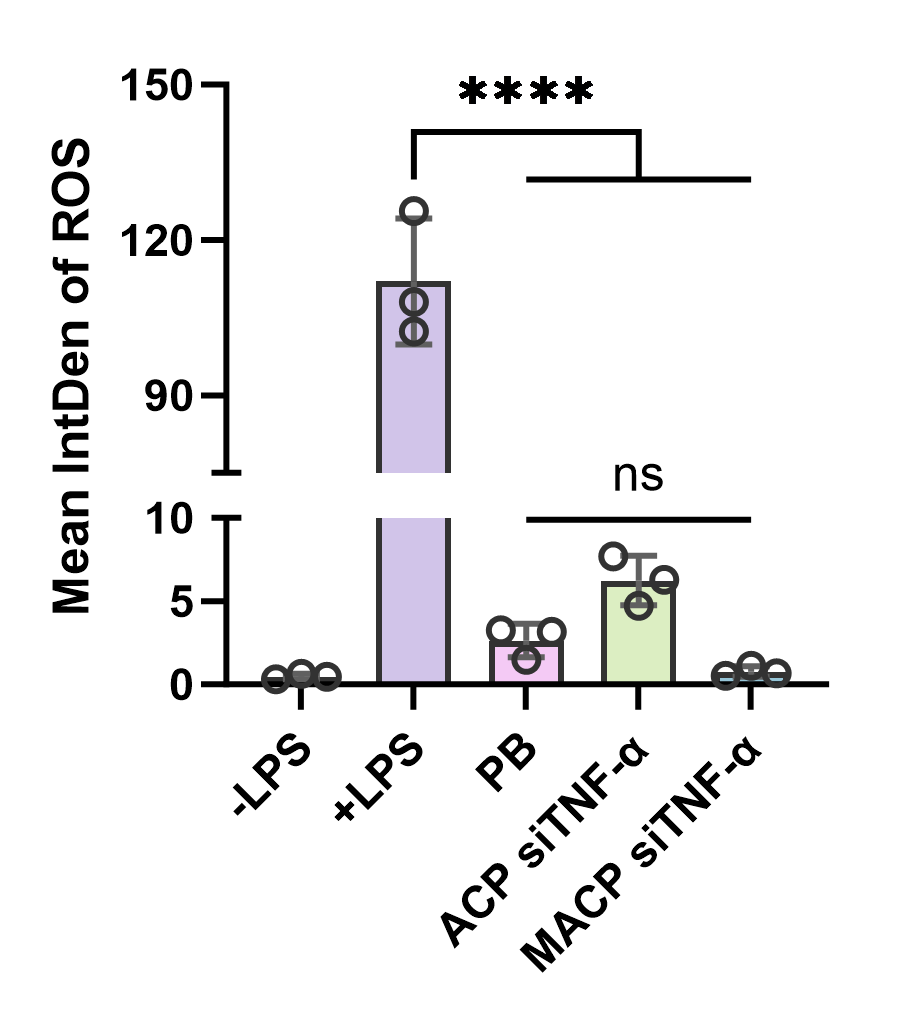


**Figure S1. Mean Intercellular Density of ROS.**

Semi-quantitative analysis of intracellular reactive oxygen species (ROS) levels across different treatment groups was performed using ImageJ software.
